# Prenatal Exposure to Modafinil and the Risk of Major Congenital Anomalies and Other Adverse Neonatal and Pediatric Outcomes

**DOI:** 10.1002/pds.70448

**Published:** 2026-08-02

**Authors:** Melinda Kanoun, Naïm Bouazza, Jean‐Marc Treluyer, Mathis Collier

**Affiliations:** ^1^ Unité de Recherche Clinique Necker Cochin AP‐HP Paris France; ^2^ Université Paris Cité, Inserm, Pharmacologie et évaluations des thérapeutiques chez l'enfant et la femme enceinte Paris France; ^3^ CIC‐1419 Inserm, Cochin‐Necker Paris France

**Keywords:** birth defects, national healthcare database, pharmacoepidemiology, pregnancy, retrospective study

## Abstract

**Objective:**

To assess the risk of major congenital anomalies (MCAs) and other adverse outcomes following prenatal exposure to modafinil.

**Methods:**

This retrospective cohort study used the French national healthcare database (SNDS) to include singleton children born between January 2009 and September 2024 of women aged 15–55 years. Prenatal exposure to psychostimulants was defined as maternal dispensation during pregnancy. Children prenatally exposed to modafinil were compared with two control groups: children prenatally exposed to methylphenidate and children prenatally unexposed to any psychostimulant matched to the exposed group using propensity‐score matching (PSM). Outcomes included MCAs, neurodevelopmental disorders (NDs), and specialized consultations. Analyses included descriptive statistics, survival analysis, regression models, and dose–response analyses.

**Results:**

Of 10 955 766 included children, 865 were prenatally exposed to modafinil and 950 to methylphenidate. Exposure to modafinil during the first trimester of pregnancy was statistically associated with increased risk of MCA compared to methylphenidate, although the confidence interval was wide and close to the null (aRR = 1.77 [1.01–3.07]). Comparison with PS‐matched unexposed population indicated a possible modest increase in risk, albeit with a wide confidence interval crossing the null (aRR = 1.23 [0.76–1.99]), showing no clear association. Occurrence of NDs (aHR = 0.96 [0.56–1.65]), and specialized consultations (aHR = 1.08 [0.91–1.28]) were similar in the modafinil and PSM unexposed groups but were higher in the methylphenidate group (NDs: aHR = 0.48 [0.29–0.80]; specialized consultations: aHR = 0.71 [0.59–0.85]).

**Conclusion:**

This study shows no increase in neurodevelopmental risk, while a moderate MCA risk cannot be excluded.

## Key Points

- This study aimed to investigate the risk of Major Congenital Anomalies (MCAs) associated with prenatal exposure to modafinil.
- Using French national administrative health data, the occurrence of MCAs was compared between children prenatally exposed to modafinil, children prenatally exposed to methylphenidate, and children exposed to no psychostimulants with comparable baseline characteristics.
- A moderate increase in MCA risk cannot be excluded; however, the absence of consistently precise estimates, a dose–response pattern, and concordant findings across comparator groups limits strong causal evidence.

## Plain Language Summary

This study looked at whether taking modafinil during pregnancy increases the risk of major birth defects or other health problems in children. We analyzed data from nearly 11 million children born in France between January 2009 and September 2024. We compared children exposed to modafinil before birth with those exposed to another drug, methylphenidate, and those not exposed to any psychostimulant but with similar characteristics. The main focus was on birth defects, but other issues such as fetal growth and neurodevelopmental issues were also analyzed. We found a slight increase in birth defects among children exposed to modafinil compared to the methylphenidate group and no increase compared to the unexposed group. Rates of neurodevelopmental issues were similar between the modafinil group and unexposed children, and actually lower than in the methylphenidate group. Overall, the findings suggest that a large increase in the risk of adverse outcomes following exposure to modafinil during pregnancy is unlikely; however, a moderate increase in risk of birth defects cannot be excluded.

## 1 Introduction

Modafinil is a psychostimulant that improves alertness and cognitive function [1]. Although the European Medicines Agency (EMA) has limited its approval to narcolepsy [2], it is approved for a variety of conditions associated with excessive daytime sleepiness (EDS), sleep apnea, and shift work disorders in other countries [3]. Typical daily doses of modafinil range from 200 to 400 mg [4].

In 2019, concerns were raised about the safety of modafinil exposure during pregnancy. Analysis of the manufacturer's pregnancy registries in the US and post‐marketing data revealed several risks to fetuses and newborns, including major congenital anomalies (MCAs), with 15%–17.3% affected infants including 4% cardiac anomalies [5, 6]. In response, the EMA and Health Canada published direct communications to healthcare professionals, warning against the use of modafinil during pregnancy.

Following warnings from health authorities, small case series were rapidly published, yielding contradictory results [7]. A subsequent analysis by Kaplan *et al.* using the US Pregnancy Registry identified MCAs in 16/119 live births (13.4%) evaluated after modafinil or armodafinil exposure [8]. A study by Damkier *et al.* based on Danish national health registries, identified MCAs in 6/49 births following exposure to modafinil during the first trimester (12.2%, aOR = 2.7, 95% CI [1.1–6.9] vs. unexposed infants) [9]. In contrast, Cesta *et al.* reported MCAs in 3/133 live births (2.6%, RR = 1.06, 95% CI [0.35–3.25] vs. unexposed infants) following exposure to modafinil during the first trimester in Swedish and Norwegian health registries [10]. These studies used MCA definitions that were close but not perfectly consistent.

Currently available data, limited and contradictory [7], do not allow a definitive assessment of the risk of MCA following prenatal exposure to modafinil. Consequently, although modafinil may be beneficial in the treatment of certain sleep disorders, it is now subject to significant restrictions and warnings, particularly with regard to its use during pregnancy. With this in mind, this study aims to investigate the risk of MCAs associated with prenatal exposure to modafinil.

## 2 Methods

### 2.1 Data Source

This retrospective cohort study was conducted using the French national administrative health data system (Système National des Données de Santé, SNDS) which covers 98.9% of the French population [11]. It is a fully anonymized database based on the national health care reimbursement database linked to the national hospital discharge database [12]. It comprises exhaustive data on patient health care expenditure such as dispensed drugs or outpatient medical care and chronic diseases. It also comprises a proxy for economic status, the affiliation to CMU‐C/C2S (*Couverture maladie universelle complémentaire/Complémentaire santé solidaire*), which is a special national insurance allowing free access to health care for people with low income. Moreover, it comprises diagnoses of all hospital admissions in France and patients' status for full reimbursement of care related to a serious and costly long‐term disease (LTD), both classified according to the International Classification of Diseases, 10th Revision (ICD‐10). Mother–child data linkage was performed using a unique mother–child identifier when available [13], or, otherwise, based on health insurance dependency status from child to mother and temporal combined with administrative concordance of the maternal delivery stay and the infant birth stay. Linkage was available for 98.5% of all births (2009–2010: 96.1%; 2011 onwards: 98.4%–99.1%). Although the causes of linkage failure could not be precisely determined, non‐linkage was more frequent for non‐live births (3.7% vs. 0.7%) and in specific hospitals, consistent with administrative data limitations. Mother–child data from the SNDS have been used previously to conduct drug safety perinatal studies [14, 15, 16, 17].

### 2.2 Study Population

All singleton children born between January 1, 2009 and September 30, 2024 of women aged between 15 and 55 years after at least 22 weeks of gestation (WG) were included. We excluded children whose birth stay could not be linked to their mother's delivery stay. We excluded all children prenatally exposed to a known or suspected teratogen [16, 18] (see Supporting Information Appendix A), to both modafinil and methylphenidate, or to other psychostimulants.

### 2.3 Drug Exposure

Exposure windows of interest were the first trimester of pregnancy (T1, defined as Days 0–91 after conception, corresponding to the main period of organogenesis) and total duration of pregnancy. Prenatal drug exposure (psychostimulants, known or suspected teratogens) was defined as having at least one maternal prescription filled during the relevant exposure window, including 1 month in the preconception period, as drug prescriptions are filled on a 30‐day basis. Cumulative modafinil dose was calculated by adding up the doses dispensed over each prescription, estimated from the number of dispensed boxes, tablets per box and dose per tablet. The average daily dose was then obtained by dividing the cumulative dose by the number of days covered [19]. Cumulative and average daily doses of modafinil were calculated over both the first trimester and the total duration of pregnancy.

### 2.4 Outcomes

The primary outcome was the diagnosis of a major congenital anomaly (MCA) as per EUROCAT classification (version 1.5), at hospital discharge after birth or up to 1 year of life [20]. Secondary outcomes were hospitalization or onset of LTD status for neurodevelopmental disorders (NDs–ICD‐10 codes F70–F98), recourse to outpatient specialized consultations (composite outcome including speech therapy, psychiatry, consultation in a medical and psychoeducational center) [15, 19], and the following perinatal outcomes restricted to live births: cesarean delivery, prematurity, defined as birth at < 37 full WG, and small‐for‐gestational‐age (SGA) status, defined as being born below the 10th percentile of weight per gestational age among all births in the SNDS (2009–September 2025).

The choice of exposure window depended on the outcome under study. For the primary outcome (MCA), exposure was assessed during the first trimester only, reflecting the etiologically relevant period for structural malformations and ensuring a fixed exposure window common to all included pregnancies. For all secondary outcomes, including perinatal and neurodevelopmental outcomes, exposure was assessed over the entire duration of pregnancy.

### 2.5 Covariates

Sociodemographic, general health, pregnancy‐ and narcolepsy‐specific baseline characteristics were assessed in the study population using the registry of health conditions of the SNDS [21], version G11, and information from the delivery stay (age, place of residence). Maternal eligibility for the CMU‐C/C2S, inflation‐corrected maternal income based on maternity allowance, and the demographic French deprivation index (FDep) [22] were also assessed as proxies for low‐income status. The FDep index is a composite score that estimates social deprivation based on the postal code of residence using median income and rates of high school graduates, workers, and unemployment. See details on covariates definitions in the Supporting Information Appendix B.

### 2.6 Statistical Analysis

Children prenatally exposed to modafinil were compared to two distinct control groups: (1) children prenatally exposed to methylphenidate, an active comparator which is not associated with overall MCA risk [23], as per Damkier *et al.*'s study design [9], and (2) children unexposed to any psychostimulant during pregnancy matched to the exposed group using propensity‐score (PS) matching. PS calculation was based on the following variables that could affect psychostimulant treatment: maternal age at delivery, FDep quintile, estimated maternal income quintile, affiliation to CMU‐C/C2S, the occurrence of a sleep study in the 2 years preceding pregnancy, and the presence of each of the following comorbidities: narcolepsy, hypertension disorder, hospitalized psychiatric disorders, and history of psychotropic treatment. PS variables were selected based on literature review and clinical expertise, subject to their availability and reliability in the SNDS. Propensity score matching was performed once, based on exposure to modafinil at any point during pregnancy. For the analysis of MCAs, the matched population was subsequently restricted to pairs in which modafinil exposure occurred during the first trimester.

Descriptive statistics were performed for each group. Quantitative data were presented as mean [standard deviation]. Differences between groups were assessed using a two‐tailed Student's *t*‐test for maternal age and birth weight and the Mann–Whitney *U* test for gestational age. Categorical variables were described by number and percentage for each modality; differences between groups were assessed using a two‐tailed chi‐squared test or a two‐tailed Fisher's exact test for variables that had five or less cases. For time‐to‐event outcomes, the analysis was based on the number of cases per person‐year (PY) to quantify occurrences, and the log‐rank test was used to compare different groups. Exposure trends over years were analyzed using linear regression. *p*‐values of 0.05 or less were considered to indicate statistical significance.

The primary outcome was analyzed using a multivariable logistic regression. Covariates included in the propensity score model were used as adjustment variables in outcome regression models, both for the active‐comparator analysis with methylphenidate and for the analysis based on the PS‐matched unexposed group, the latter thus relying on a doubly robust estimation strategy. NDs and specialized consultations were separately analyzed using survival analysis with Kaplan–Meier curves and a Cox proportional hazards regression adjusted on the same covariates to estimate the risk of NDs in liveborn children prenatally exposed to modafinil versus the two control groups. Children were followed up from birth until outcome occurrence, death from any cause, or September 30, 2025, whichever came first.

A dose–response analysis was also carried out, focusing on adjusted risks of MCA, NDs, and specialized consultations as a function of dispensed modafinil dose during corresponding exposure windows. This analysis was based on low/high average daily dose of modafinil on the one hand, and low/high cumulated dose on the other hand. Daily modafinil dose was categorized as low for a < 300 mg/day regimen [4], and cumulated modafinil dose was categorized as low if it was strictly less than the median value among the included patients.

A sensitivity analysis for the primary outcome (MCA) restricted to infants born to women who had received at least two dispensations of psychostimulants during the first trimester of pregnancy was performed. Pregnancies with prenatal psychostimulant exposure not fulfilling this criterion were excluded from this sensitivity analysis. Again, the matched population was subsequently restricted to pairs in which two prescriptions of modafinil were dispensed during the first trimester of pregnancy, based on the PS matching performed in the main analysis.

To explore the potential impact of differential exposure opportunity related to gestational duration, a second sensitivity analysis was performed restricting the population to term births. MCAs were not studied in this analysis, as exposure for this outcome was defined during the first trimester of pregnancy, a fixed window common to all pregnancies included in the study. Moreover, prematurity could be a collider variable between psychostimulant exposure and MCA, leading to potentially biased estimates when using prematurity as an exclusion criterion.

Data extraction was performed with SAS Enterprise Guide v. 8.3.7 software (SAS Institute, Cary, NC, USA). Data analysis and graphics were made with R v. 4.3.3 statistical software and MS Office software.

## 3 Results

### 3.1 Patient Inclusion and Baseline Characteristics

**FIGURE 1:**
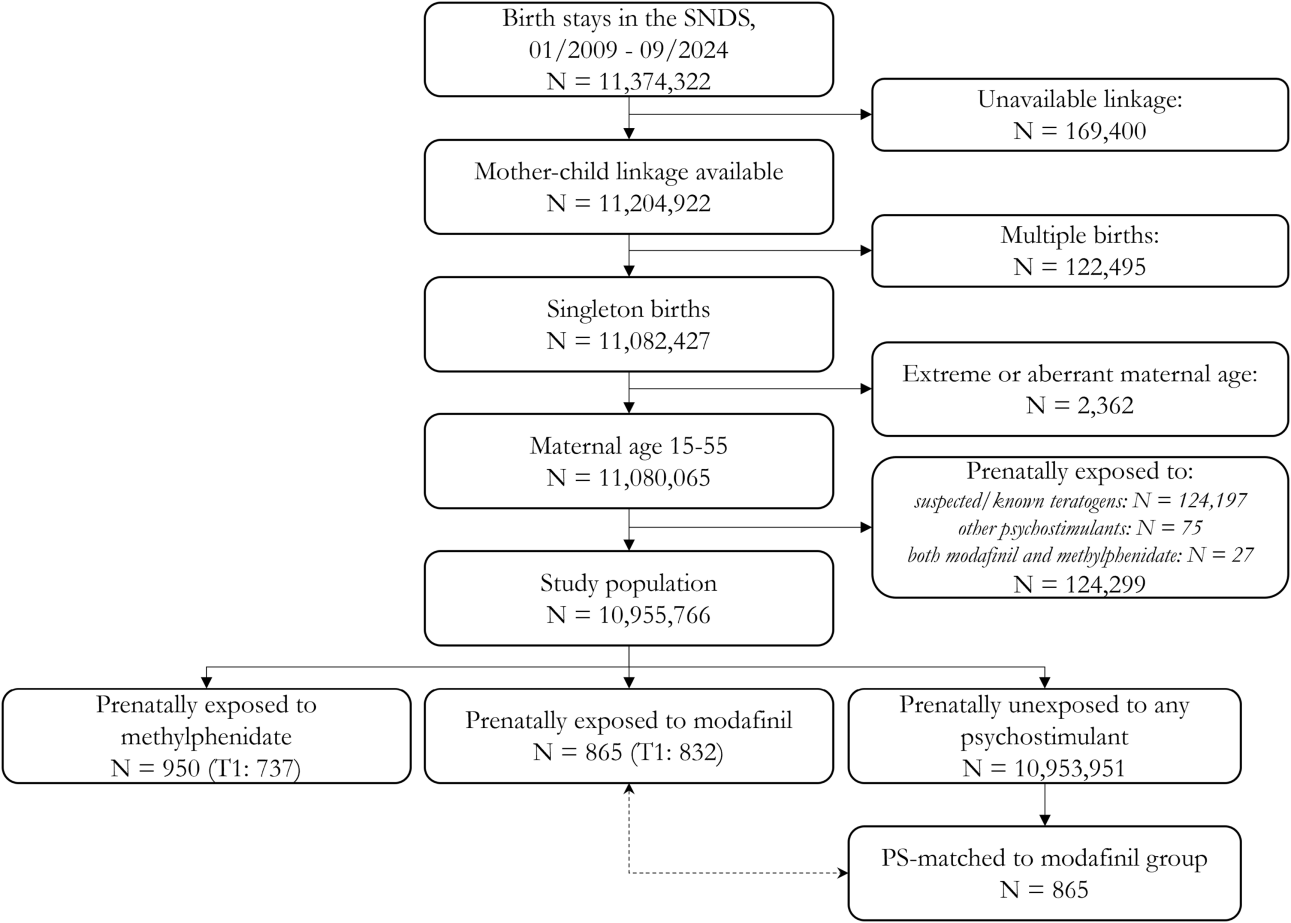
Study flowchart. SNDS, Système National des Données de Santé; PS, propensity score; T1, first trimester of pregnancy.

The cohort included 10 955 766 patients, 865 of which were prenatally exposed to modafinil and 950 to methylphenidate (see Figure 1). A total of 865 prenatally unexposed patients were PS‐matched to the modafinil group with a standardized mean difference (SMD) in propensity score below 0.001 (see Supporting Information Appendix C).

Cohort baseline characteristics are reported in Table 1. Children prenatally exposed to modafinil were born of women younger in age than in the methylphenidate group (mean age: 31.2 vs. 32.6 years, *p* < 0.001), in a less fragile socioeconomic situation (CMU‐C/C2S affiliation 5.5% vs. 21.2%, *p* < 0.001, favorable income quintiles distribution, *p* < 0.001) and who were less likely to suffer from psychiatric comorbidities (history of hospitalized psychiatric disorder 1.6% vs. 4.2%, *p* = 0.002, history of psychotropic treatment 16.5% vs. 28.3%, *p* < 0.001) and gestational diabetes (10.5% vs. 15.6%, *p* = 0.004). Maternal history of narcolepsy and sleep studies were however more frequent in the modafinil group (28.7% vs. 6.9%, *p* < 0.001 and 23.5% vs. 6.4%, *p* < 0.001, respectively), reflecting the expected indication for this medication. Modafinil and PS‐matched groups only differed in rates of maternal preexisting diabetes (0.5% vs. 1.5% respectively, *p* = 0.048) and gestational diabetes (10.5% vs. 15.0% respectively, *p* = 0.006). Prenatal exposure occurred at least during the first trimester of the pregnancy for 832 children in the modafinil group (96.2%) and 737 children in the methylphenidate group (77.6%).

**TABLE 1:** Cohort baseline characteristics.

Prenatal exposure	Modafinil		Methylphenidate		Unexposed	PS‐matched unexposed		*p*_1_	*p*_2_
Exposure at	Any point	T1	Any point	T1	—	All pairs	T1 pairs		
Number of births (*N*)	*N* = 865	*N* = 832	*N* = 950	*N* = 737	*N* = 10 953 951	*N* = 865	*N* = 832		
Maternal age at delivery (years)									
Mean [SD]	31.2 [4.8]	31.2 [4.8]	32.6 [5.5]	32.1 [5.6]	30.2 [5.4]	31.3 [4.9]	31.3 [4.8]	< 0.001***	0.866
< 25	65 (7.5)	60 (7.2)	79 (8.3)	72 (9.8)	1 640 149 (15.0)	64 (7.4)	59 (7.1)		
25–29	253 (29.2)	246 (29.6)	162 (17.1)	134 (18.2)	3 348 073 (30.6)	247 (28.6)	239 (28.7)		
30–34	325 (37.6)	315 (37.9)	324 (34.1)	268 (36.4)	3 613 924 (33.0)	339 (39.2)	330 (39.7)		
≥ 35	222 (25.7)	211 (25.4)	385 (40.5)	263 (35.7)	2 351 805 (21.5)	215 (24.9)	204 (24.5)		
Low‐income status									
CMU‐C/C2S	48 (5.5)	45 (5.4)	201 (21.2)	138 (18.7)	1 787 542 (16.3)	56 (6.5)	53 (6.4)	< 0.001***	0.479
Household social deprivation index (FDep)									
Q1 (most favored)	185 (21.4)	180 (21.6)	222 (23.4)	193 (26.2)	2 089 610 (19.1)	180 (20.8)	176 (21.2)	0.132	1
Q2	181 (20.9)	171 (20.6)	200 (21.1)	154 (20.9)	2 084 855 (19.0)	180 (20.8)	172 (20.7)		
Q3	159 (18.4)	152 (18.3)	201 (21.2)	148 (20.1)	2 094 955 (19.1)	163 (18.8)	158 (19.0)		
Q4	170 (19.7)	161 (19.4)	183 (19.3)	140 (19.0)	2 088 057 (19.1)	169 (19.5)	157 (18.9)		
Q5 (least favored)	151 (17.5)	149 (17.9)	128 (13.5)	89 (12.1)	2 094 421 (19.1)	154 (17.8)	150 (18.0)		
Unknown	19 (2.2)	19 (2.3)	16 (1.7)	13 (1.8)	502 053 (4.6)	19 (2.2)	19 (2.3)		
Maternal estimated monthly income									
Q1 income (€0–€1825)	88 (10.2)	85 (10.2)	96 (10.1)	71 (9.6)	1 227 199 (11.2)	88 (10.2)	85 (10.2)	< 0.001***	0.996
Q2 income (€1826–€2286)	105 (12.1)	102 (12.3)	96 (10.1)	74 (10.0)	1 227 172 (11.2)	105 (12.1)	102 (12.3)		
Q3 income (€2287–€2745)	95 (11.0)	93 (11.2)	104 (10.9)	87 (11.8)	1 227 182 (11.2)	99 (11.4)	96 (11.5)		
Q4 income (€2746–€3504)	122 (14.1)	117 (14.1)	103 (10.8)	89 (12.1)	1 227 159 (11.2)	118 (13.6)	114 (13.7)		
Q5 income (> €3504)	154 (17.8)	152 (18.3)	124 (13.1)	98 (13.3)	1 227 116 (11.2)	147 (17.0)	146 (17.5)		
No income found	301 (34.8)	283 (34.0)	427 (44.9)	318 (43.1)	4 818 123 (44.0)	308 (35.6)	289 (34.7)		
Maternal medical history									
Hypertension	25 (2.9)	25 (3.0)	28 (2.9)	23 (3.1)	121 032 (1.1)	27 (3.1)	27 (3.2)	1	0.888
Diabetes	4 (0.5)	4 (0.5)	12 (1.3)	9 (1.2)	69 332 (0.6)	13 (1.5)	12 (1.4)	0.079	0.048*
Psychiatric disorder	14 (1.6)	12 (1.4)	40 (4.2)	36 (4.9)	29 706 (0.3)	9 (1.0)	8 (1.0)	0.002**	0.401
Psychotropic treatment	143 (16.5)	139 (16.7)	270 (28.4)	233 (31.6)	396 016 (3.6)	144 (16.6)	141 (16.9)	< 0.001***	1
Multiple sclerosis	5 (0.6)	5 (0.6)	4 (0.4)	3 (0.4)	9115 (0.1)	2 (0.2)	1 (0.1)	0.745	0.452
Narcolepsy	248 (28.7)	235 (28.2)	66 (6.9)	64 (8.7)	1298 (< 0.1)	248 (28.7)	235 (28.2)	< 0.001***	1
Sleep study	203 (23.5)	196 (23.6)	61 (6.4)	59 (8.0)	21 911 (0.2)	209 (24.2)	201 (24.2)	< 0.001***	0.778
Pregnancy‐related characteristics									
Gestational diabetes	91 (10.5)	87 (10.5)	148 (15.6)	111 (15.1)	1 344 494 (12.3)	130 (15.0)	127 (15.3)	0.004**	0.006**
Preeclampsia	22 (2.5)	20 (2.4)	27 (2.8)	22 (3.0)	208 639 (1.9)	22 (2.5)	21 (2.5)	0.986	1
Folic acid suppl.	384 (44.4)	367 (44.1)	436 (45.9)	342 (46.4)	4 137 928 (37.8)	382 (44.2)	372 (44.7)	0.615	0.961

*Note: p*_1_: Modafinil vs. Methylphenidate, *p*_2_: Modafinil vs. PS‐matched unexposed. *p < 0.05, **p < 0.01, ***p < 0.001. *p*‐values are based on exposure at any point during pregnancy.

Abbreviations: CMU‐C/C2S, *Couverture maladie universelle complémentaire/Complémentaire santé solidaire*; FDep, French social deprivation index; LTD, long‐term disease; PS, propensity score; Q1–Q5, quintiles 1–5 of the distribution; SD, standard deviation; T1, first trimester of pregnancy.

**FIGURE 2:**
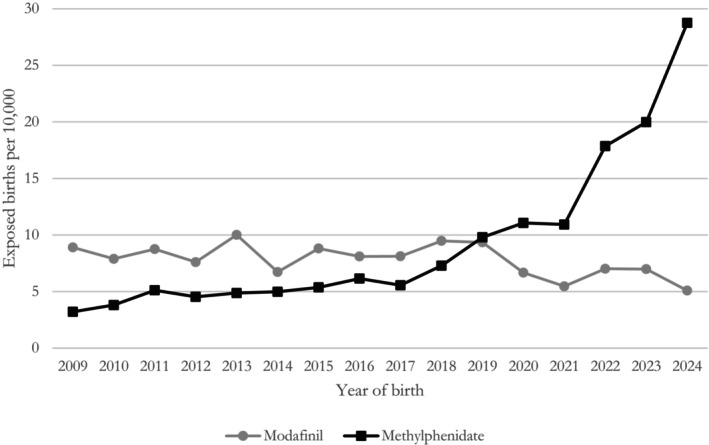
Trends of prenatal psychostimulants exposure over inclusion period.

Proportion of pregnancies exposed to psychostimulants in monotherapy each year is shown in Figure 2. Prenatal exposure to modafinil was slightly decreasing over the inclusion period (*p* = 0.021) while prenatal exposure to methylphenidate showed a clear increasing trend (*p* < 0.001).

### 3.2 Main and Secondary Outcomes Analysis

**FIGURE 3:**
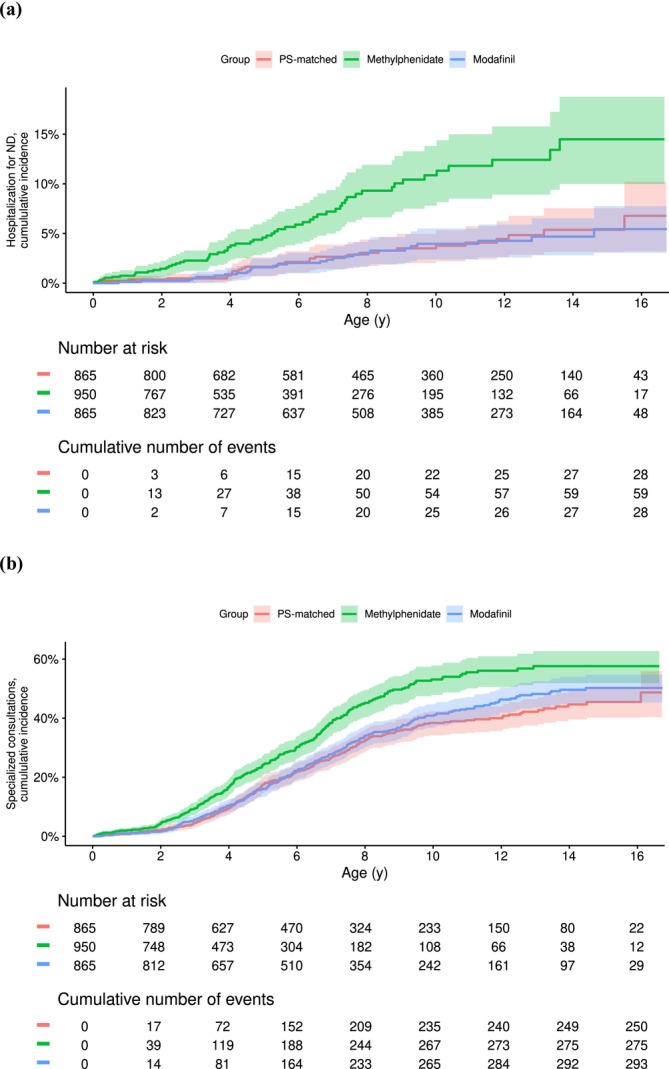
Kaplan–Meier survival curves, first occurrence of neurodevelopmental disorder (a) and first recourse to an outpatient specialized consultation (b) based on exposure at any point during pregnancy. PS, propensity score; ND, neurodevelopmental disorder; y, years.

Prevalence of analyzed outcomes are reported in Tables 2 and 3. Based on first‐trimester exposure, there were 35 cases of MCA in the modafinil group (4.2%), 21 in the methylphenidate group (2.8%), and 29 in the PS‐matched unexposed group (3.5%), yielding no statistically significant difference regardless of the control group considered (*p* = 0.190 and *p* = 0.524, respectively). Based on exposure at any point during pregnancy and among live births, gestational age was higher in the modafinil group than in the methylphenidate group (39.2 vs. 38.8 WG, *p* < 0.001) and similar to that of the PS‐matched group (39.2 vs. 39.1 WG, *p* = 0.564). Rate of prematurity was higher in the methylphenidate group and did not significantly differ in the PS‐matched group (4.7% vs. 7.9%, *p* = 0.008 and 4.7% vs. 5.5%, *p* = 0.205, respectively). Likewise, cesarean delivery rate was higher in the methylphenidate group (21.9% vs. 26.5%, *p* = 0.036) and similar in the PS‐matched group (21.9% vs. 21.4%, *p* = 0.840). There were no significant differences in rates of SGA. Incidence of NDs and specialized consultations were lower in the modafinil group compared to the methylphenidate group overall (*p* < 0.001 for both) but were similar to that of the PS‐matched group (*p* = 0.793 and *p* = 0.284, respectively). Corresponding Kaplan–Meier survival curves are shown in Figure 3.

**TABLE 2:** Birth outcomes based on first‐trimester exposure.

Prenatal exposure	Modafinil (T1)	Methylphenidate (T1)	Unexposed	PS‐matched unexposed (T1 pairs)	*p*_1_	*p*_2_
Number of births (*N*)	*N* = 832	*N* = 737	*N* = 10 953 951	*N* = 832		
Issue						
Live birth	820 (98.6)	728 (98.8)	10 871 565 (99.2)	822 (98.8)	0.836	0.829
Stillbirth	8 (1.0)	5 (0.7)	49 832 (0.5)	8 (1.0)		
Medical termination	4 (0.5)	4 (0.5)	32 554 (0.3)	2 (0.2)		
MCA	35 (4.2)	21 (2.8)	328 784 (3.0)	29 (3.5)	0.190	0.524

*Note: p*_1_: Modafinil vs. Methylphenidate, *p*_2_: Modafinil vs. PS‐matched unexposed. *p < 0.05, **p < 0.01, ***p < 0.001.

Abbreviations: MCA, major congenital anomaly; PS, propensity score; T1, first trimester of pregnancy.

**TABLE 3:** Obstetric, fetal, and pediatric outcomes based on exposure at any point during pregnancy, restricted to live births.

Prenatal exposure	Modafinil (whole pregnancy)	Methylphenidate (whole pregnancy)	Unexposed	PS‐matched unexposed (all pairs)	*p*_1_	*p*_2_
Number of live births (*N*)	*N* = 853	*N* = 941	*N* = 10 871 565	*N* = 855		
Delivery						
Cesarean section	187 (21.9)	249 (26.5)	2 163 749 (19.9)	183 (21.4)	0.029**	0.840
Gestational age, mean [SD]	39.2 [1.7]	38.8 [1.9]	39.1 [1.8]	39.1 [2.0]	< 0.001***	0.564
Prematurity (< 37 WG)	40 (4.7)	74 (7.9)	601 179 (5.5)	53 (6.2)	0.008**	0.205
Preterm (32–36 WG)	36 (4.2)	67 (7.1)	515 196 (4.7)	44 (5.1)		
Very preterm (28–31 WG)	2 (0.2)	4 (0.4)	56 792 (0.5)	5 (0.6)		
Extremely preterm (< 28 WG)	2 (0.2)	3 (0.3)	29 191 (0.3)	4 (0.5)		
Infant characteristics						
Sex M	458 (53.7)	489 (52.0)	5 554 645 (51.1)	444 (51.9)	0.494	0.496
Birth weight (g), mean [SD]	3307 [490]	3265 [541]	3283 [522]	3272 [538]	0.086	0.163
SGA	75 (8.8)	80 (8.5)	1 065 007 (9.8)	80 (9.4)	0.893	0.748
ND [IRR/1000PY]						
Any ND	28 [3.5]	59 [10.3]	277 330 [2.9]	28 [3.8]	< 0.001***	0.793
Intellectual disability	3 [0.4]	8 [1.3]	38 957 [0.4]	4 [0.5]	0.053	0.624
Disorders of psych. development	23 [2.9]	41 [7.1]	184 219 [1.9]	16 [2.1]	< 0.001***	0.365
Behavioral and emotional disorders	12 [1.5]	29 [4.9]	115 827 [1.2]	16 [2.1]	< 0.001***	0.335
Specialized consultations [IRR/1000PY]						
Any specialized consultation	293 [44.8]	275 [57.1]	2 763 923 [33.0]	251 [40.4]	< 0.001***	0.284
Speech therapy	260 [38.4]	233 [46.6]	2 515 292 [29.4]	231 [36.2]	< 0.001***	0.597
Psychiatrist	71 [9.3]	83 [14.8]	484 497 [5.0]	49 [6.7]	< 0.001***	0.094
Psychoeducational center	3 [0.4]	3 [0.5]	45 836 [0.5]	5 [0.7]	0.623	0.416

*Note: p*_1_: Modafinil vs. Methylphenidate, *p*_2_: Modafinil vs. PS‐matched unexposed. *p < 0.05, **p < 0.01, ***p < 0.001.

Abbreviations: IRR/1000PY, incidence rate ratio per 1000 persons‐years; ND, neurodevelopmental disorder; PS, propensity score; SD, standard deviation; SGA, small for gestational age; WG, weeks of gestation.

**FIGURE 4:**
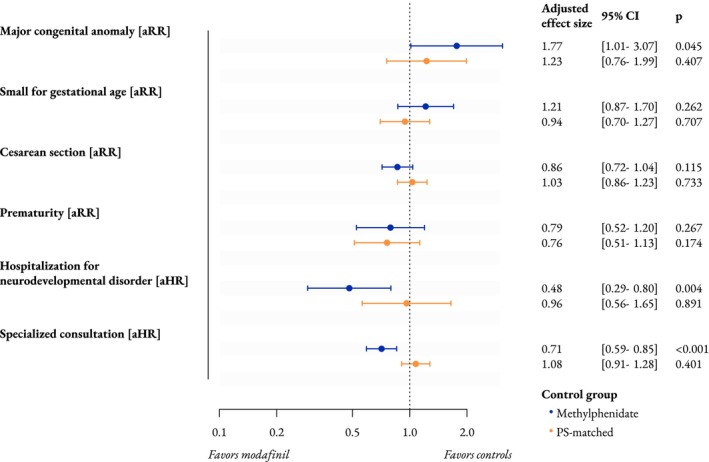
Adjusted risk ratios and hazard ratios of outcomes. Risk of major congenital anomaly based on first‐trimester exposure; risk of other outcomes based on exposure at any point during pregnancy. CI, confidence interval; aRR, adjusted risk ratio; aHR, adjusted hazard ratio; PS, propensity score.

Adjusted risk ratios (aRR) and hazard ratios (aHR) are shown in Figure 4. Crude risk and hazard ratios (RR, HR) and crude and adjusted odds ratios (OR, aOR) were also calculated for the purpose of comparison with other studies (see Supporting Information Appendix C). Prenatal exposure to modafinil during the first trimester of pregnancy was statistically associated with an increased risk of MCA in comparison with methylphenidate (aRR = 1.77, 95% CI [1.01–3.07]). This association was not found in comparison with the PS‐matched population (aRR = 1.23 [0.76–1.99]). No clear association was found between prenatal exposure to modafinil at any point during pregnancy and cesarean delivery (methylphenidate: aRR = 0.86 [0.72–1.04], PS‐matched: aRR = 1.03 [0.86–1.23]) or SGA status (methylphenidate: aRR = 1.21 [0.87–1.70], PS‐matched: aRR = 0.94 [0.70–1.27]), with estimates differing by comparator and confidence intervals crossing the null. For prematurity, point estimates were consistently below unity in both comparisons (methylphenidate: aRR = 0.79 [0.52–1.20]; PS‐matched: aRR = 0.76 [0.51–1.13]), although confidence intervals included the null. Compared to the modafinil group, NDs and specialized consultations were significantly higher in the methylphenidate group (aHR = 0.48 [0.29–0.80] and aHR = 0.71 [0.59–0.85], respectively) and similar in the modafinil and PS‐matched groups (aHR = 0.96 [0.56–1.65] and aHR = 1.08 [0.91–1.28], respectively).

### 3.3 Dose–Response Analysis

Modafinil daily dose and cumulative dose distributions across included patients are shown in the Supporting Information Appendix D. Median cumulative modafinil dose dispensed to exposed mothers through pregnancy was 9.6 g. Population characteristics and outcomes for children prenatally exposed to low‐dose versus high‐dose modafinil are also reported in the Supporting Information Appendix D. Of 865 children prenatally exposed to modafinil, 484 (56.0%) were exposed to a daily dose greater than or equal to 300 mg/day and 435 (50.3%) to a cumulative dose greater than or equal to 9.6 g. High versus low daily dose was not associated with higher occurrence of MCA (aRR = 0.66 [0.34–1.28]), ND (aHR = 1.14 [0.52–2.52]) or specialized consultations (aHR = 0.72 [0.31–1.67]). High versus low cumulated dose during the first trimester was not associated with MCA (aRR = 0.92 [0.48–1.75]); high versus low cumulated dose during the total duration of pregnancy was likewise not associated with ND (aHR = 0.72 [0.31–1.67]) nor specialized consultations (aHR = 0.91 [0.72–1.15]).

### 3.4 Sensitivity Analysis

A total of 405 children were born to women with at least two dispensations of modafinil during the first trimester, and 380 to women with at least two dispensations of methylphenidate during the first trimester. Supporting Information Tables E1 and E2, corresponding to Tables 1 and 2 recomputed with the new exposure criterion, show the characteristics of this restricted population. Sensitivity analysis yielded MCA aRRs of 1.72 [0.75–3.93] compared to children prenatally exposed to methylphenidate and 1.39 [0.63–3.10] compared to PS‐matched unexposed children, i.e., similar point estimates as the main analysis but with larger confidence intervals.

Among 10 285 471 infants born at term, 813 were prenatally exposed to modafinil and 870 to methylphenidate at any point during pregnancy. Based on the PS matching performed in the main analysis, 806 infants among the unexposed matched population were born at term. Regression models yielded similar results as the main analysis. Detailed results are shown in the Supporting Information Tables F1 and F2, which correspond to Tables 1 and 3 in the main analysis.

## 4 Discussion

This large retrospective cohort study, based on French health insurance data, revealed that prenatal exposure to modafinil during the first trimester was associated with a higher estimated risk of MCAs when compared with prenatal exposure to methylphenidate, with an adjusted risk ratio of 1.77 and a lower confidence bound marginally above unity. In contrast, comparison with a PS‐matched unexposed population yielded a more modest point estimate above unity but with a confidence interval crossing the null, compatible with both no association and a moderate increase in risk. Sensitivity analysis restricted to 2+ dispensations yielded stable point estimates but with wider confidence intervals, probably reflecting limited precision rather than evidence of effect modification. No clear dose–response pattern was identified.

These results are partially consistent with some previous studies, while diverging on certain points. Kaplan *et al.* [8] reported a markedly higher MCA rate of 13.4% among live births using MACDP definitions [24] in a hybrid prospective and retrospective cohort. Outcome definition, the absence of a control group, and the pooling of modafinil and armodafinil inside a single exposure group may explain the differences with this study (armodafinil is not prescribed in France). Damkier *et al.* [9] reported a 12.2% rate of MCA in pregnancies exposed to modafinil using EUROCAT definitions, with an aOR of 3.4, 95% CI [1.2–9.7] compared to methylphenidate, and an aOR of 2.7 [1.1–6.9] compared to unexposed pregnancies. However, this study was based on a limited cohort of 49 pregnancies on modafinil, with six cases of MCA, resulting in a wide confidence interval. Comparison with unexposed pregnancies could suffer from residual confounding since it did not take sociodemographic factors into account and was carried out using plain logistic regression adjustment as opposed to more robust methods (matching, IPTW, etc.). In contrast, this study includes a substantially larger number of exposed pregnancies while reporting considerably lower rates of MCA (4.2%), closer to the results of Cesta *et al.* [10]. The latter reported an MCA rate of 2.6% and a crude relative risk (RR) close to unity, albeit with wide confidence intervals (RR = 1.06 [0.35–3.25]). Taken together, these findings indicate that a moderate increase in MCA risk cannot be excluded; however, the available evidence lacks several features that would support a stronger causal interpretation, including a dose–response relationship and estimates both clearly separated from the null and concordant across comparator groups.

No clear association was found between prenatal exposure to modafinil and cesarean section or small‐for‐gestational age status, consistent across comparator groups and dose–response/sensitivity analyses. Estimates for prematurity were lower among modafinil‐exposed pregnancies versus both comparator groups. However, the fact that confidence intervals overlapped the null and the absence of an established physiological rationale for a true protective effect of modafinil on prematurity precludes firm conclusions.

We observed no clear association between prenatal exposure to modafinil and risk of NDs or recourse to specialized consultations compared to controls, consistently with the results of Madsen *et al.* [25]. In an apparent contradiction with the latter, the occurrence of NDs and specialized consultations in the methylphenidate group was higher than in the general population, even after adjustment for both lower socioeconomic status and psychiatric history, which are strong confounding factors for these outcomes. This might be explained by unmeasured residual confounding, pediatric exposure to psychotropic drugs, genetic predispositions which were not accessible in the SNDS, and timing of exposure during pregnancy. Indeed, in a recent study, Suarez *et al.* studied the risk of NDs in children prenatally exposed to methylphenidate versus prenatally unexposed children using PS‐weighted analysis [26]. They found an increased adjusted risk of NDs associated with methylphenidate early prenatal exposure (aHR = 1.16 [1.05–1.27]) and a similar estimate for late pregnancy exposure with reduced precision (aHR = 1.15 [0.97–1.36]).

This study has several strengths. The use of data from the SNDS, covering the French population with quasi‐exhaustivity, allows the creation of a large cohort and thus high statistical power and generalization of the results to a large population. Additionally, the SNDS offers detailed data on healthcare, expenditures, diagnoses, and medications dispensed, enabling in‐depth analysis of risk factors. However, this study also has limitations. As an observational study, there is a risk of residual confounding bias. Although we adjusted for several covariates, other unmeasured factors such as alcohol, smoking, or other environmental exposures during pregnancy could influence the results. Additionally, exposure to modafinil and methylphenidate is based on reimbursement data, which may not perfectly reflect actual consumption of the drug, although sensitivity analysis based on infants born of women who received two prescriptions of psychostimulants probably mitigates this bias. Finally, diagnosis data was limited to hospital admissions and LTD status, and outpatient diagnoses were as such unavailable for inclusion, outcome, and covariate assessment.

In conclusion, this nationwide cohort study does not provide evidence of a large increase in the risk of adverse perinatal and neurodevelopmental outcomes following prenatal exposure to modafinil; however, a moderate increase in MCA risk remains plausible given the observed point estimate and associated uncertainty. Moreover, the clear increasing trend of prenatal methylphenidate exposure and the potential associated risk of NDs calls for caution and continued research.

## Author Contributions

Study design: Mathis Collier and Jean‐Marc Treluyer. Data extraction: Melinda Kanoun and Mathis Collier. Data analysis: Melinda Kanoun and Mathis Collier. Data interpretation: Naïm Bouazza and Jean‐Marc Treluyer. Draft the manuscript: Melinda Kanoun. Critically revised the manuscript: Melinda Kanoun, Naïm Bouazza, and Jean‐Marc Treluyer.

## Funding

The authors have nothing to report.

## Ethics Statement

Access to the SNDS was granted to the authors by French decree no. 2021–848. All data used were deidentified. Therefore, no informed consent from the patients was required. The Strengthening the Reporting of Observational Studies in Epidemiology (STROBE) reporting guideline was followed.

## Conflicts of Interest

The authors declare no conflicts of interest.

## Supporting information


**Appendix A.** Known or suspected teratogens.
**Appendix B**. Variables definition.
**Appendix C**. Main analysis.
**Appendix D**. Dose–response analysis.
**Appendix E**. First sensitivity analysis (at least two dispensations during the first trimester of pregnancy).
**Appendix F**. Second sensitivity analysis (restricted to term births).

## Data Availability

The original contributions in this analysis are included in this published article and its Supporting Information files. The authors have full access to all data in the analysis and take full responsibility for the integrity, accuracy, and conduct of the research. Code is available upon reasonable request to the authors.

## References

[1] D. Banerjee , M. V. Vitiello , and R. R. Grunstein , “Pharmacotherapy for Excessive Daytime Sleepiness,” Sleep Medicine Reviews 8 (2004): 339–354, 10.1016/j.smrv.2004.03.002. 15336235

[2] D. Hilton‐Jones , M. Bowler , H. Lochmueller , et al., “Modafinil for Excessive Daytime Sleepiness in Myotonic Dystrophy Type 1–The Patients' Perspective,” Neuromuscular Disorders 22 (2012): 597–603, 10.1016/j.nmd.2012.02.005. 22425060

[3] T. Roth , J. R. L. Schwartz , M. Hirshkowitz , M. K. Erman , J. M. Dayno , and S. Arora , “Evaluation of the Safety of Modafinil for Treatment of Excessive Sleepiness,” Journal of Clinical Sleep Medicine 3 (2007): 595–602. 17993041 PMC2045706

[4] P. Tanayapong , V. Tantrakul , S. Liamsombut , et al., “Comparative Efficacy and Safety of Multiple Wake‐Promoting Agents for the Treatment of Residual Sleepiness in Obstructive Sleep Apnea Despite Continuous Positive Airway Pressure: A Systematic Review and Network Meta‐Analysis of Randomized Controlled Trials,” CNS Drugs 39 (2025): 527–544, 10.1007/s40263-025-01175-7. 40208562 PMC12058958

[5] Government of Canada HC , “ALERTEC (Modafinil) and the Risk of Congenital Anomalies–Recalls, Advisories and Safety Alerts—Canada.Ca [Internet],” Government of Canada, Health Canada, Marketed Health Products, 2021, https://recalls‐rappels.canada.ca/en/alert‐recall/alertec‐modafinil‐and‐risk‐congenital‐anomalies.

[6] European Medicines Agency , “PRAC Recommendations on Signals,” 2025, https://www.ema.europa.eu/en/documents/prac‐recommendation/prac‐recommendations‐signals‐adopted‐8‐11‐april‐2019‐prac‐meeting_en.pdf.

[7] B. Marin , I. Arnulf , M. Latour , et al., “Potential Teratogenicity of Modafinil ‐ Conflicting Evidence, Need for Research,” Gynecologie Obstetrique Fertilite et Senologie 51 (2023): 186–189, 10.1016/j.gofs.2023.01.003. 36642328

[8] S. Kaplan , D. L. Braverman , I. Frishman , and N. Bartov , “Pregnancy and Fetal Outcomes Following Exposure to Modafinil and Armodafinil During Pregnancy,” JAMA Internal Medicine 181 (2021): 275–277, 10.1001/jamainternmed.2020.4009. 33074297 PMC7573789

[9] P. Damkier and A. Broe , “First‐Trimester Pregnancy Exposure to Modafinil and Risk of Congenital Malformations,” Journal of the American Medical Association 323 (2020): 374–376, 10.1001/jama.2019.20008. 31990303 PMC6990936

[10] C. E. Cesta , A. Engeland , P. Karlsson , H. Kieler , J. Reutfors , and K. Furu , “Incidence of Malformations After Early Pregnancy Exposure to Modafinil in Sweden and Norway,” Journal of the American Medical Association 324 (2020): 895–897, 10.1001/jama.2020.9840. 32870289 PMC7489822

[11] P. Tuppin , J. Rudant , P. Constantinou , et al., “Value of a National Administrative Database to Guide Public Decisions: From the système national d'information interrégimes de l'Assurance Maladie (SNIIRAM) to the système national des données de santé (SNDS) in France,” Revue d'Épidémiologie et de Santé Publique 65, no. Suppl 4 (2017): S149–S167, 10.1016/j.respe.2017.05.004. 28756037

[12] J. Bezin , M. Duong , R. Lassalle , et al., “The National Healthcare System Claims Databases in France, SNIIRAM and EGB: Powerful Tools for Pharmacoepidemiology,” Pharmacoepidemiology and Drug Safety 26 (2017): 954–962, 10.1002/pds.4233. 28544284

[13] P.‐O. Blotière , A. Weill , M. Dalichampt , et al., “Development of an Algorithm to Identify Pregnancy Episodes and Related Outcomes in Health Care Claims Databases: An Application to Antiepileptic Drug Use in 4.9 Million Pregnant Women in France,” Pharmacoepidemiology and Drug Safety 27 (2018): 763–770, 10.1002/pds.4556. 29763992 PMC6055607

[14] M. Bourdon , P. Santulli , N. Beeker , et al., “Impact of Clomiphene Citrate on Multiple Gestation Births and Perinatal Outcomes: A Nationwide Cohort Study,” Fertility and Sterility 124 (2025): 334–343, 10.1016/j.fertnstert.2025.04.005. 40222700

[15] M. Collier , L. Chouchana , P. Frange , et al., “Risk of Neurodevelopmental Disorders in Children Exposed to Human Immunodeficiency Virus and Antiretrovirals in Utero: A National Cohort Study in France,” Clinical Infectious Diseases 81 (2025): 92–100, 10.1093/cid/ciae610. 39905585

[16] L. Chouchana , M. Collier , C. Martin , P.‐R. Burgel , and J.‐M. Treluyer , “CFTR Modulators and Pregnancy Outcomes: Early Findings From a Nationwide Cohort Study,” Journal of Cystic Fibrosis 24 (2025): 469–475, 10.1016/j.jcf.2025.03.002. 40058987

[17] M. Tisseyre , M. Collier , N. Beeker , F. Kaguelidou , J.‐M. Treluyer , and L. Chouchana , “Prenatal Exposure to Proton Pump Inhibitors and Risk of Serious Infections in Offspring During the First Year of Life: A Nationwide Cohort Study,” Drug Safety 48 (2025): 265–277, 10.1007/s40264-024-01496-4. 39630354

[18] M. Louchet , M. Collier , N. Beeker , et al., “Trends in Harmful Drug Exposure During Pregnancy in France Between 2013 and 2019: A Nationwide Cohort Study,” PLoS One 19 (2024): e0295897, 10.1371/journal.pone.0295897. 38198446 PMC10781191

[19] J. Coste , P.‐O. Blotiere , S. Miranda , et al., “Risk of Early Neurodevelopmental Disorders Associated With in Utero Exposure to Valproate and Other Antiepileptic Drugs: A Nationwide Cohort Study in France,” Scientific Reports 10 (2020): 17362, 10.1038/s41598-020-74409-x. 33093466 PMC7581762

[20] A. Kinsner‐Ovaskainen , M. Lanzoni , E. Garne , et al., “A Sustainable Solution for the Activities of the European Network for Surveillance of Congenital Anomalies: EUROCAT as Part of the EU Platform on Rare Diseases Registration,” European Journal of Medical Genetics 61 (2018): 513–517, 10.1016/j.ejmg.2018.03.008. 29597096

[21] A. Rachas , C. Gastaldi‐Ménager , P. Denis , et al., “The Economic Burden of Disease in France From the National Health Insurance Perspective: The Healthcare Expenditures and Conditions Mapping Used to Prepare the French Social Security Funding Act and the Public Health Act,” Medical Care 60 (2022): 655–664, 10.1097/MLR.0000000000001745. 35880776 PMC9365254

[22] C. Pornet , C. Delpierre , O. Dejardin , et al., “Construction of an Adaptable European Transnational Ecological Deprivation Index: The French Version,” Journal of Epidemiology and Community Health 66 (2012): 982–989, 10.1136/jech-2011-200311. 22544918 PMC3465837

[23] K. F. Huybrechts , G. Bröms , L. B. Christensen , et al., “Association Between Methylphenidate and Amphetamine Use in Pregnancy and Risk of Congenital Malformations: A Cohort Study From the International Pregnancy Safety Study Consortium,” JAMA Psychiatry 75 (2018): 167–175, 10.1001/jamapsychiatry.2017.3644. 29238795 PMC5838573

[24] A. Correa‐Villaseñor , J. Cragan , J. Kucik , L. O'Leary , C. Siffel , and L. Williams , “The Metropolitan Atlanta Congenital Defects Program: 35 Years of Birth Defects Surveillance at the Centers for Disease Control and Prevention,” Birth Defects Research Part A: Clinical and Molecular Teratology 67 (2003): 617–624, 10.1002/bdra.10111. 14703783

[25] M. G. Madsen , J. L. Zhu , T. Munk‐Olsen , et al., “Prevalence and Temporal Trends of Attention Deficit Hyperactivity Disorder Medication Fills During Pregnancy and Breastfeeding in Denmark,” Paediatric Drugs 27 (2025): 233–246, 10.1007/s40272-024-00671-5. 39806199 PMC11829916

[26] E. A. Suarez , B. T. Bateman , S. Hernandez‐Diaz , et al., “Prescription Stimulant Use During Pregnancy and Risk of Neurodevelopmental Disorders in Children,” JAMA Psychiatry 81 (2024): 477–488, 10.1001/jamapsychiatry.2023.5073. 38265792 PMC10809143

