# Supplementary material for: Prenatal Exposure to Modafinil and the Risk of Major Congenital Anomalies and Other Adverse Neonatal and Pediatric Outcomes
Source: Pharmacoepidemiol Drug Saf. 2026 Aug 2;35(8):e70448. doi: 10.1002/pds.70448 (PMC13429282; doi:10.1002/pds.70448)
Supplement: Supplementary file 1 — Appendix A. Known or suspected teratogens. Appendix B. Variables definition. Appendix C. Main analysis. Appendix D. Dose–response analysis. Appendix E. First sensitivity analysis (at least two dispensations during the first trimester of pregnancy). Appendix F. Second sensitivity analysis (restricted to term births). [file PDS-35-e70448-s001.docx]

**Electronic Supplementary Material**

**Title:**

Prenatal Exposure to Modafinil and the Risk of Major Congenital Anomalies and Other Adverse Neonatal and Pediatric Outcomes

**Authors:**

**Melinda Kanoun^1 2^, Naïm Bouazza^1 2 3^, Jean-Marc Treluyer^1 2 3^, Mathis Collier^1 2^**

^1^ Unité de Recherche Clinique Necker Cochin, AP-HP, France

^2^ Université Paris Cité, Inserm, Pharmacologie et évaluations des thérapeutiques chez l'enfant et la femme enceinte, F-75006, Paris, France

^3^ CIC-1419 Inserm, Cochin-Necker, Paris, France

**Corresponding author:**

Mathis Collier

Unité de Recherche Clinique Necker Cochin, AP-HP

Paris, France

[mathis.collier@aphp.fr](mailto:mathis.collier@aphp.fr)

# [Appendix](https://www.ncbi.nlm.nih.gov/pmc/articles/PMC10781191/) A: Known or suspected teratogens

**Supplementary table A1**

| **Therapeutic group** | **ATC code** | **International nonproprietary names** |
| --- | --- | --- |
| **Systemic retinoids** | D10BA01 | isotretinoin |
|  | D11AH04 | alitretinoin |
|  | D05BB02 | acitretin |
| **Anticonvulsivants** | N03AF01 | carbamazepin |
|  | N03AG01 | valproic acid |
|  | N03AB02 | phenytoin |
|  | N03AB05 | fosphenytoin |
|  | N03AB52 | phenytoin, combinations |
|  | N03AA01 | phenobarbital |
|  | N03AA03 | primidone |
|  | N03AX11 | topiramate |
| **Antithyroid drugs** | H03BB02 | thiamazole |
|  | H03BB01 | carbimazole |
|  | H03BA02 | propylthiouracil |
| **Anticoagulants** | B01AA03 | warfarin |
|  | B01AA07 | acenocoumarol |
|  | B01AA12 | fluindione |
| **Antibiotics** | J01GA01 | streptomycin |
|  | J04AM01 | streptomycin and isoniazid |
|  | J01EA01 | trimethoprime |
|  | J02AC01 | fluconazole |
| **Ergot alkaloids** | N02CA01 | dihydroergotamine |
|  | N02CA51 | dihydroergotamine, combinations |
|  | N02CA02 | ergotamine |
|  | N02CA52 | ergotamine, combinations excl. psycholeptics |
|  | N02CA72 | ergotamine, combinations with psycholeptics |
| **Androgens** | G03BA03 | testosterone |
|  | G03XA01 | danazol |
| **Alkylating agents** | L01A |  |
| **Antimetabolites** | L01B |  |
| **Other antineoplastic agents** | L01X |  |
| **Others** | A02BB01 | misoprostol |
|  | M01AE56 | naproxen and misoprostol |
|  | N06BA01 | amfetamine |
|  | L04AX02 | thalidomide |
|  | L04AX03 | methotrexate |
|  | G03CB02 | diethystilbestrol |
|  | L04AA06 | mycophenolate |
|  | L04AA13 | leflunomide |
|  | L04AA31 | teriflunomide |
|  | N05AN01 | lithium |

List of known or suspected teratogens. ATC Anatomic-Therapeutic-Chemical classification

# [Appendix](https://www.ncbi.nlm.nih.gov/pmc/articles/PMC10781191/) B: Variables definition

**Supplementary table B1**

| **Variable** | **Codes** | **Code type** | **Time frame** | **Comment** |
| --- | --- | --- | --- | --- |
| Fetal exposure to psychostimulants | Modafinil: N06BA07  Methylphenidate: N06BA04  Pitolisant: N07XX11  Solriamfetol: N06BA14  [ATC]  Sodium oxybate: 9281199, 9454694, 9455802 [UCD-7] | ATC, UCD-7 | Dispensing from 30 days before conception to delivery (whole pregnancy window) or 91 days after conception (T1 window) | Psychostimulants are dispensed on a 30-day basis |
| History of diabetes | Diabetes: E10-14 [ICD-10, hospitalization and LTD status]  Diabetes complication: G590, G632, G730, G990, H280, H360, I792, L97, M142, M146, N083 [ICD-10]  Antidiabetics: A10A, A10BA, A10BB, A10BC, A10BD, A10BF, A10BG, A10BH, A10BJ01, A10BJ02, A10BJ05, A10BJ06, A10BX except A10BX06, A10X [ATC]  Antidiabetics: 3400927892912 (Jardiance^®^ 25mg) [CIP-13] | ICD-10  ATC  CIP-13 | During the year preceding conception | Diabetes as LTD status in the year preceding conception  OR hospitalization with diabetes as main discharge code in the 2 years preceding conception  OR hospitalization with diabetes complication as main discharge code and diabetes as associated code in the 2 years preceding conception  OR dispensing of 3 small packs or 2 packs (including at least one large pack) of antidiabetics, at distinct dates, in the year preceding conception or in the year before |
| History of hypertension | C02AB02, C02AC01, C02AC02, C02AC05, C02AC06, C02CA01, C02CA06, C02DC01, C02LA01, C03AA01, C03AA03, C03BA04, C03BA10, C03BA11, C03BX03, C03CA01, C03CA02, C03CA03, C03DA01, C03DB01, C03EA01, C03EA04, C07AA02, C07AA03, C07AA05, C07AA06, C07AA12, C07AA15, C07AA16, C07AA23, C07AB02, C07AB03, C07AB04, C07AB05, C07AB07, C07AB08, C07AB12, C07AG01, C07BA02, C07BB02, C07BB03, C07BB07, C07BB12, C07CA03, C07DA06, C07FB02, C07FB03, C08CA01, C08CA02, C08CA03, C08CA04, C08CA05, C08CA08, C08CA09, C08CA11, C08CA13, C08CX01, C08DA01, C08DB01, C08GA02, C09AA01, C09AA02, C09AA03, C09AA04, C09AA05, C09AA06, C09AA07, C09AA08, C09AA09, C09AA10, C09AA13, C09AA15, C09AA16, C09BA01, C09BA02, C09BA03, C09BA04, C09BA05, C09BA06, C09BA07, C09BA09, C09BA15, C09BB02, C09BB04, C09BB07, C09BB10, C09BX02, C09CA01, C09CA02, C09CA03, C09CA04, C09CA06, C09CA07, C09CA08, C09DA01, C09DA02, C09DA03, C09DA04, C09DA06, C09DA07, C09DA08, C09DB01, C09DB02, C09DB04, C09XA02, C09XA52, C10BX03 | ATC | During the year preceding conception | Dispensing of 3 small packs or 2 packs including at least one large pack, at distinct dates |
| History of psychiatric disorders | Psychotic disorders, Neurotic and mood disorders, Mental deficiency, Addictive disorders, Psychiatric disorders starting in childhood or Other psychiatric disorders |  |  |  |
| Psychotic disorders | Psychiatric disorders: F20-F22, F23-F29 [ICD-10]  Antipsychotics: N05A except N05AN01, N05AL06 [ATC]  3400932896332 (Neuriplege^®^) [CIP-13] | ICD-10  ATC  CIP-13 | ICD-10 codes: LTD status in the year before conception  OR hospitalization in the 2 years preceding conception with diagnosis as main discharge code (general hospitals) / any discharge code (psychiatric hospitals)  OR [any type of discharge code in the two years preceding pregnancy AND multiple dispensations of antipsychotics in the year preceding conception] | For each drug class: dispensing of 3 small packs or 2 packs including at least one large pack, at distinct dates |
| Neurotic and mood disorders | F30-F48 [ICD-10]  Antidepressants and mood stabilizers: N06A except levotonine (CIP-13: 3400933338022), N03AG02, N05AN01[ATC]  3400934876233, 3400934876691, 3400935444271 (Dépakote^®^) [CIP-13] | ICD-10  ATC  CIP-13 | ICD-10 codes: LTD status in the year before conception  OR hospitalization in the 2 years preceding conception with diagnosis as main discharge code (general hospitals) / any discharge code (psychiatric hospitals)  OR [any type of discharge code in the two years preceding conception AND multiple dispensations of antidepressants in the year preceding conception] |  |
| Mental deficiency | F70-F79 [ICD-10] | ICD-10 | ICD-10 codes: LTD status in the year before conception  OR hospitalization in the 2 years preceding conception with diagnosis as main discharge code (general hospitals) / any discharge code (psychiatric hospitals) |  |
| Addictive disorders | F10-F19 [ICD-10]  Nicotine substitutes: N07BA  Bupropione: N06AX12 | ICD-10  ATC | ICD-10 codes: LTD status in the year before conception  OR hospitalization in the 2 years preceding conception with diagnosis as main discharge code (general hospitals) / any discharge code (psychiatric hospitals)  OR multiple dispensations of nicotine substitutes or bupropione in the year preceding conception |  |
| Psychiatric disorders starting in childhood | F80-F98 [ICD-10] |  | ICD-10 codes: LTD status in the year before conception  OR hospitalization in the 2 years preceding conception with diagnosis as main discharge code (general hospitals) / any discharge code (psychiatric hospitals) |  |
| Other psychiatric disorders | F04, F050, F058, F059, F06, F07, F09, F23, F50-69, F99 |  | ICD-10 codes: LTD status in the year before conception  OR hospitalization in the 2 years preceding conception with diagnosis as main discharge code (general hospitals) / any discharge code (psychiatric hospitals  OR hospitalization in a psychiatric hospital without ICD-10 codes F, B220 or G20, and without detected Psychotic disorders, Neurotic and mood disorders, Mental deficiency, Addictive disorders, Psychiatric disorders starting in childhood, in the year preceding conception |  |
| History of psychotropic treatment | Antidepressants and mood stabilizers: N06A except levotonine (CIP-13: 3400933338022), N03AG02, N05AN01[ATC]  3400934876233, 3400934876691, 3400935444271 (Dépakote®) [CIP-13]  Neuroleptics: N05AN01, N05AL06 [ATC]  3400932896332 (Neuriplege^®^) [CIP-13]  Anxiolytics: N05BA01, N05BA04, N05BA05, N05BA06, N05BA08, N05BA09, N05BA11, N05BA12, N05BA16, N05BA18, N05BA21, N05BA23, N05BB01, N05BB02, N05BC01, N05BE01, N05BX03 [ATC]  Hypnotics: N05BC51, N05CB02, N05CD02, N05CD03, N05CD04, N05CD05, N05CD06, N05CD07, N05CD11, N05CF01, N05CF04, N05CF02, N05CM11, N05CM16, N05CX [ATC] | ATC  CIP-13 | During the year preceding conception | For each drug class: dispensing of 3 small packs or 2 packs including at least one large pack, at distinct dates |
| Multiple sclerosis | G35 [ICD-10]  L03AB07, L03AB08, L03AB13, L03AX13, L04AA23, L04AA27, L04AA31, L04AA36, L04AA40, L04AA50, L04AA52, L04AX07, L04AX09, N07XX07, N07XX09 [ATC] | ICD-10  ATC | ICD-10 codes: LTD status in the year before conception or hospitalization in the two years preceding conception  Drugs: during the year preceding conception | ICD-10: Hospitalization or LTD status  Drugs: Dispensing of 3 packs at distinct dates |
| Narcolepsy | G474, G479 | ICD-10 | In the two years preceding conception | Hospitalization or LTD status |
| Sleep study | 23K02Z [GHM]  AMQP012, AMQP013, AMQP015 [CCAM] | GHM  CCAM | In the two years preceding conception |  |
| Gestational diabetes | Gestational diabetes: O24 [ICD-10]  Insulins and analogues: A10A [ATC]  Blood glucose measuring devices: 1198033, 6187270, 6169480, 6188068, 6186230, 6110149, 6113490, 6115566, 6162465, 6112125, 6169310, 6112250, 6124594, 6126890, 6131602, 6177640, 6185450, 6185466, 6184640, 6185124, 6110824, 6110072, 6110563, 6111367, 1172861, 1108350, 6174735, 1167498, 1173487, 6166109, 1136894, 6188051, 6145254, 6186224, 6113426, 6115520, 6112912, 6166115, 6116637, 6126920, 6174758, 6185420, 6185443, 6179595, 6184679, 6110818, 6111350, 1186722, 6187264, 6187590, 6155620, 6111752, 6148726, 6113461, 6112639, 6162459, 6112183, 6124602, 6126937, 6131588, 6177691, 6185414, 6184662, 6185093, 6110089, 1180441, 1187408, 6111730, 1101720, 6129143, 6114897, 6187287, 6186260, 6111769, 6192762, 6113395, 6115508, 6112898, 6112154, 6112190, 6124341, 6126819, 6131594, 6177656, 6178963, 6185495, 6184633, 6111380 [LPP] | ICD-10  ATC  LPP | During pregnancy | Without diabetes status before pregnancy. |
| Preeclampsia | O14 | ICD-10 | During pregnancy | Hospitalization only |
| Folic acid supplementation during 1^st^ trimester | B03AD, B03AE01, B03AE02, B03BB | ATC | From 30 days before conception to 91 days after conception |  |
| Cesarean section | JQGA002, JQGA003, JQGA004, JQGA005 | CCAM | During delivery stay |  |
| Small for gestational age | Newborn weight according to gestational age | - |  | ≤ 10^th^ percentile |
| Neurodevelopmental disorders | Intellectual disability: F70-F79  Disorders of psychological development: F80-F89  Behavioral and emotional disorders with onset usually occurring in childhood and adolescence: F90-F98 | ICD-10 |  | Hospitalization or LTD status |
| Specialized consultations | Speech therapy:  3132, 3136, 3137, 3142 [social security act code]  AMO [outpatient act code]  Psychiatrist:  1102, 1113, 1118, 1216, 2380, 2381, 2382, 2383, 2384, 2385, 2386, 2387 [social security act code]  APY, CNP, CNF F, CNP N, CNPSY, AVY  Psychoeducational center:  2336 [social security act code] | social security act code  outpatient act code |  |  |

List of codes used in the study. ATC Anatomic-Therapeutic-Chemical classification, UCD-7 *Unité commune de dispensation*, ICD-10 International classification of diseases, 10^th^ revision, CIP-13 *Code identifiant de présentation*, CCAM *Classification commune des actes médicaux*, LPP *Liste des produits et prestations*, GHM *Groupe homogène de malades*, LTD Long-term disease

# [Appendix](https://www.ncbi.nlm.nih.gov/pmc/articles/PMC10781191/) C: Main analysis

**Supplementary figure C1**


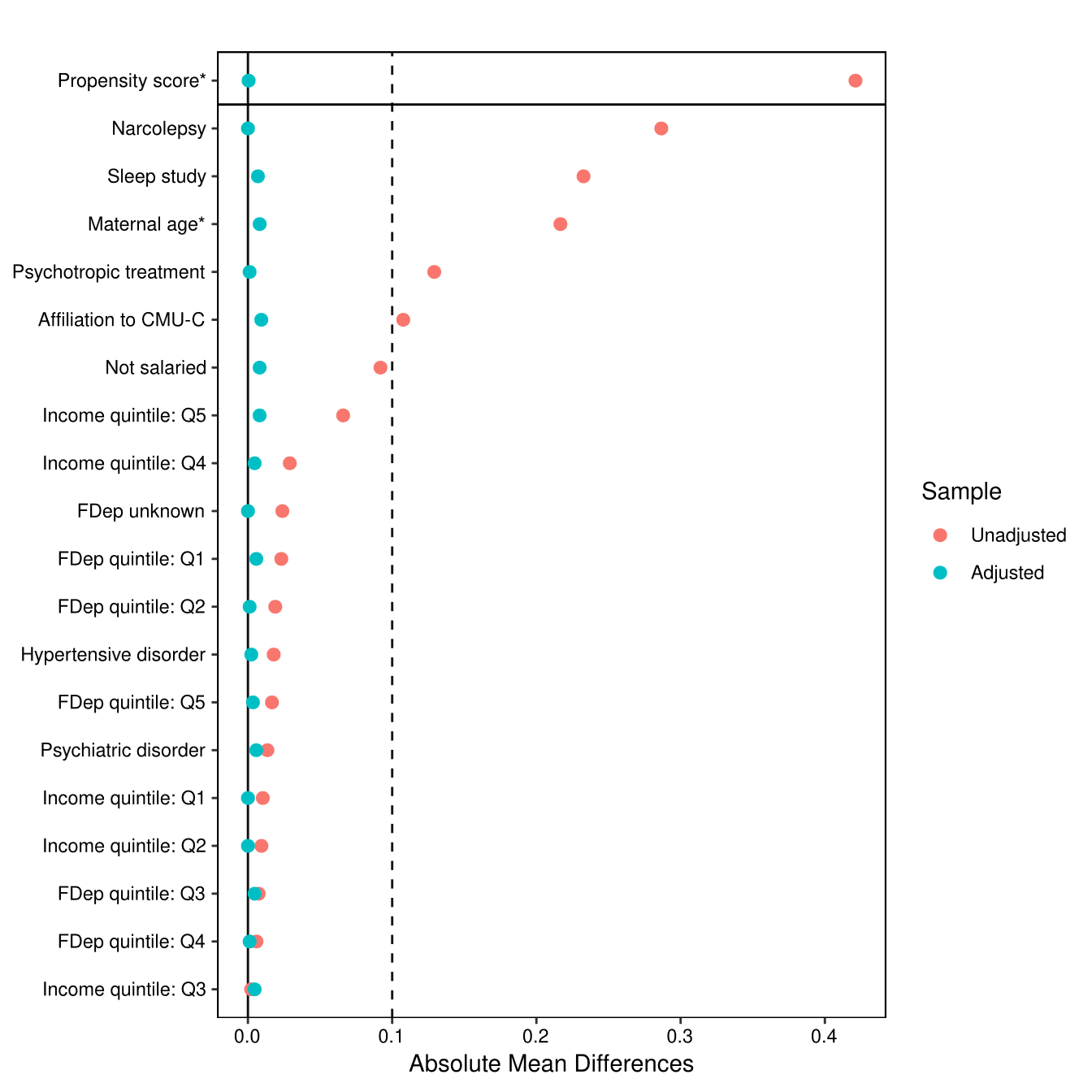


Maternal baseline covariate imbalance between children prenatally exposed to modafinil and children unexposed to psychostimulants, based on exposure at any point during pregnancy. CMU-C/C2S Couverture maladie universelle complémentaire / Complémentaire santé solidaire, FDep French social deprivation index, Q1-Q5 quintiles 1-5 of the distribution

^*^ Mean differences have been standardized

**Supplementary figure C2**


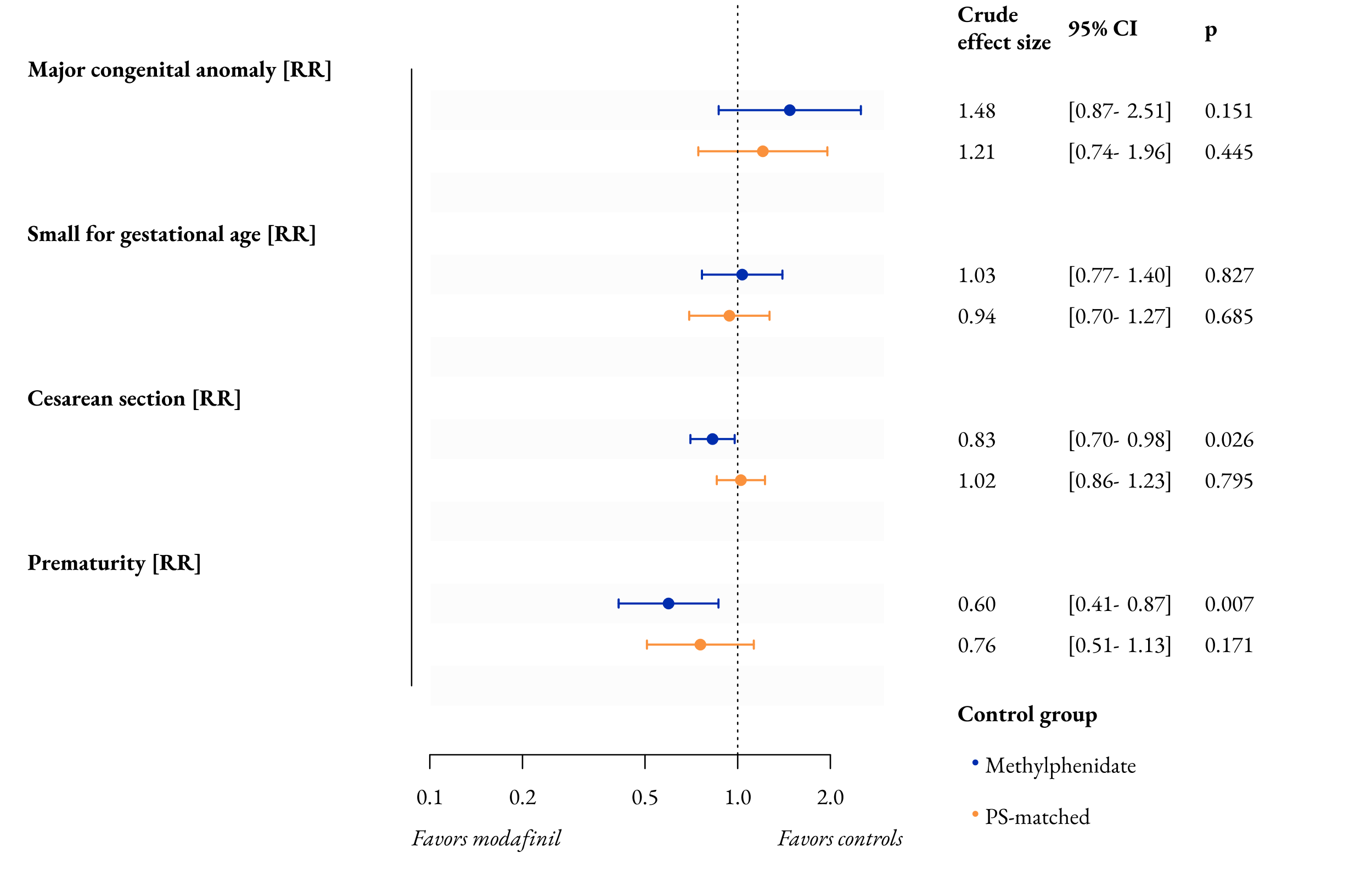


Crude risk ratios and hazard ratios of outcomes. CI confidence interval, RR risk ratio, HR hazard ratio, PS propensity score

**Supplementary figure C3**


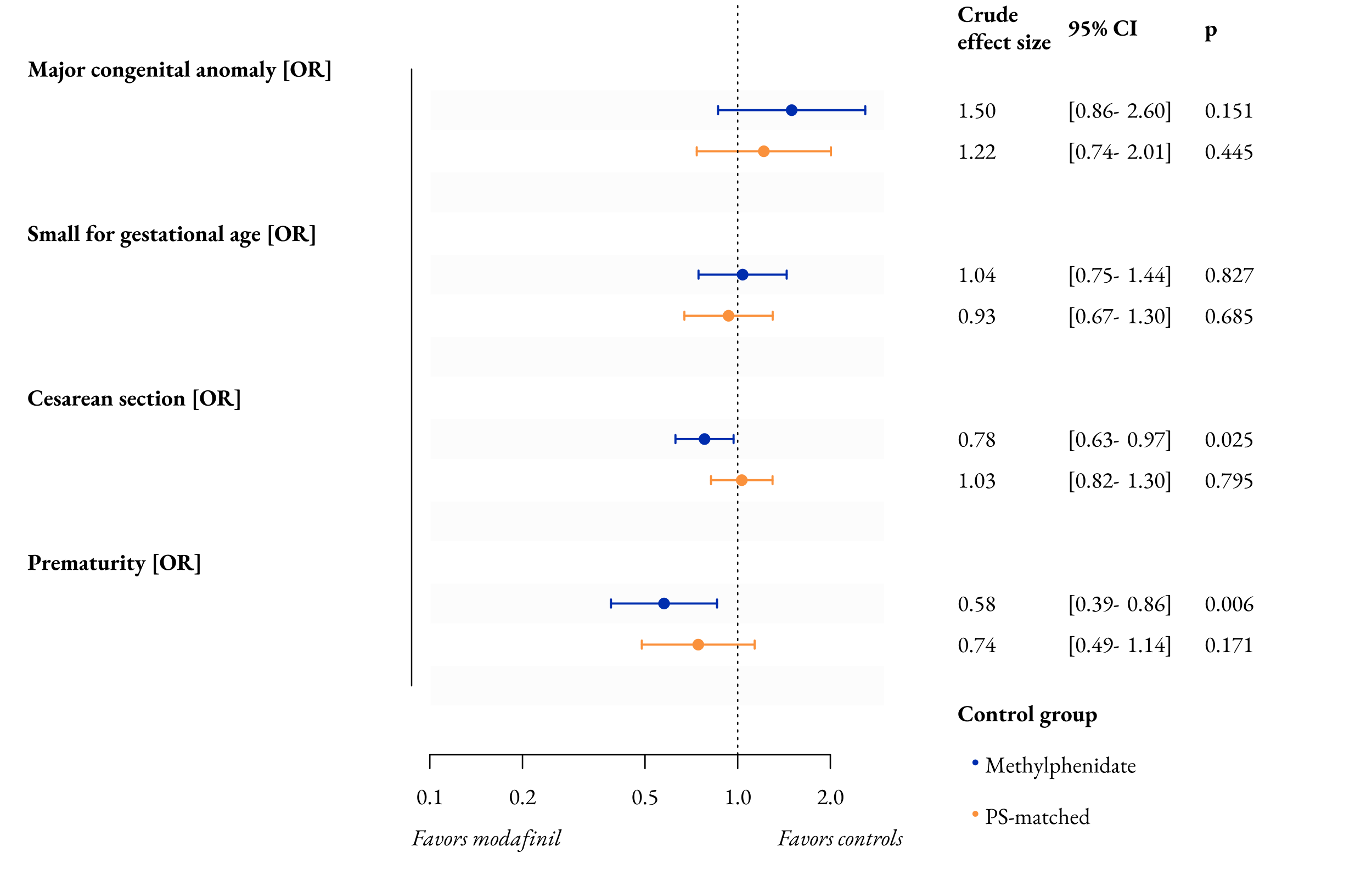


Crude odds ratios of outcomes. CI confidence interval, OR odds ratio, PS propensity score

**Supplementary figure C4**


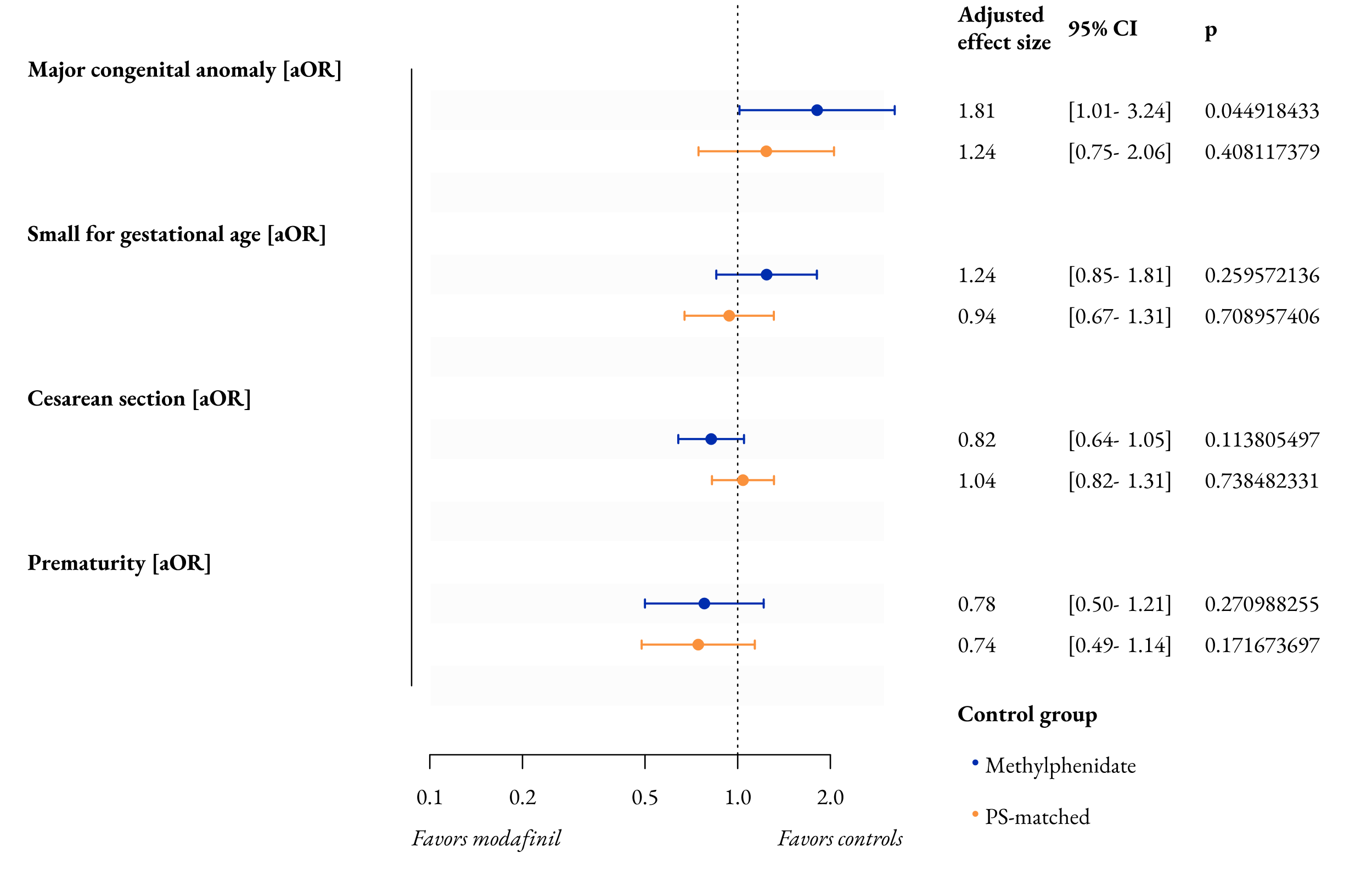


Adjusted odds ratios of outcomes. CI confidence interval, OR odds ratio, PS propensity score

# [Appendix](https://www.ncbi.nlm.nih.gov/pmc/articles/PMC10781191/) D: Dose-response analysis

**Supplementary figure D1**


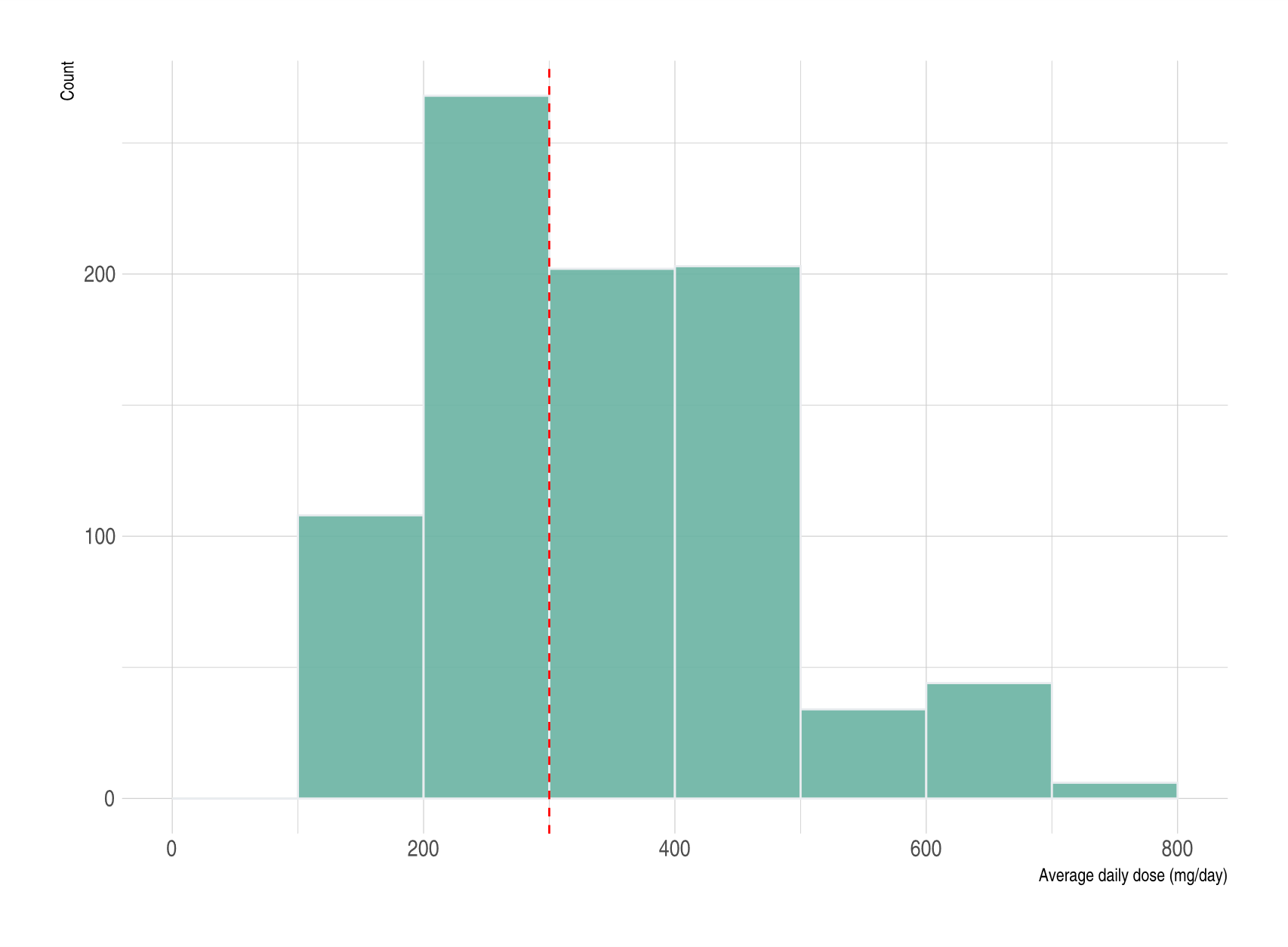


Average daily dose distribution across included patients. Intervals are left-closed. Dotted red line represents the chosen 300 mg/day threshold above which average daily dose is categorized as high

**Supplementary figure D2**


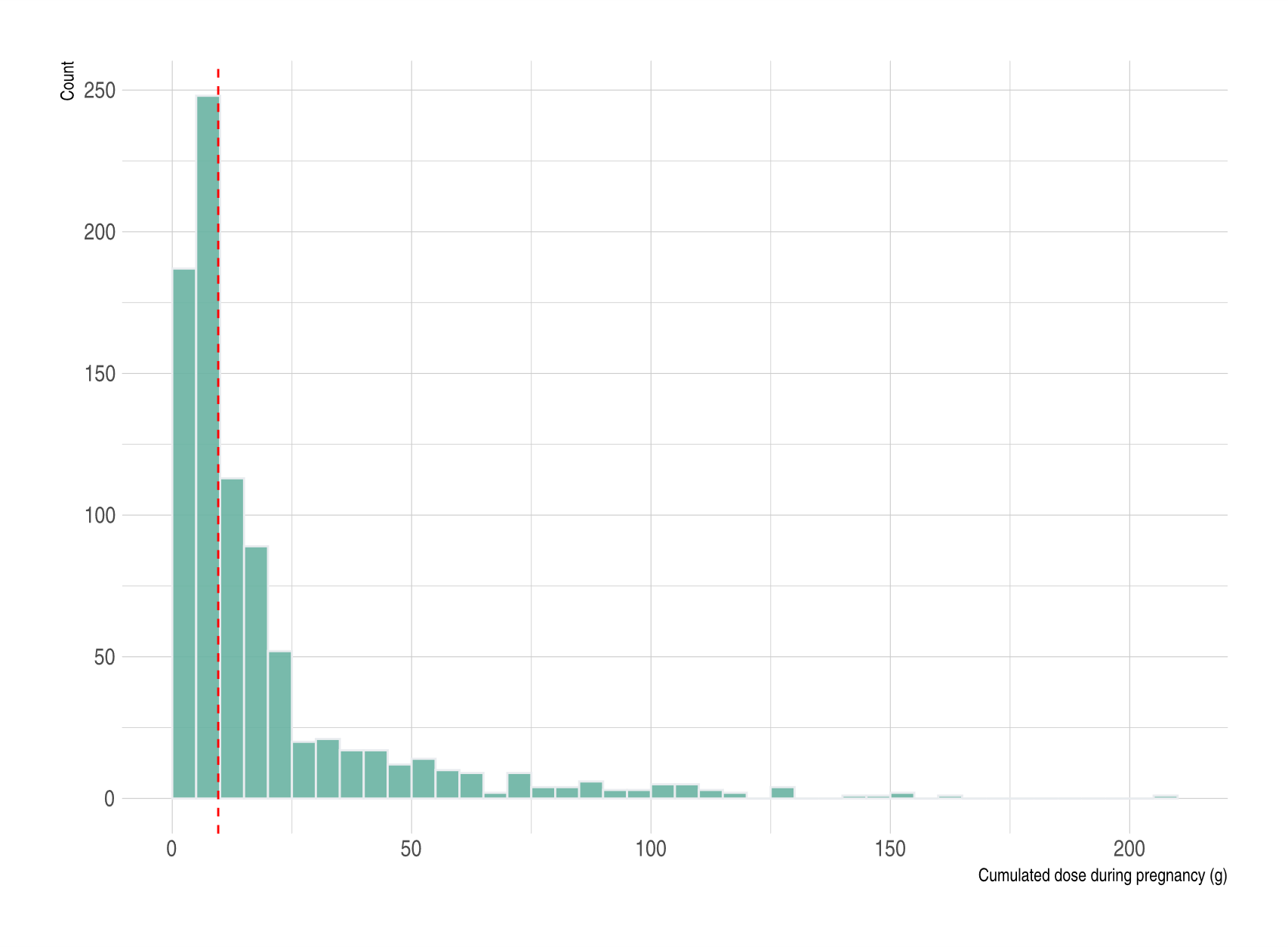


Cumulated dose distribution during pregnancy across included patients. Intervals are left-closed. Dotted red line represents the median of the distribution (9.6 g), above which cumulated dose is categorized as high

**Supplementary table D1**

|  | Prenatal exposure | Low DD modafinil  < 300 mg/day | | High DD modafinil  ≥ 300 mg/day | | p | Low CD modafinil  < 9.6 g | | High CD modafinil  ≥ 9.6 g | | p |
| --- | --- | --- | --- | --- | --- | --- | --- | --- | --- | --- | --- |
|  | Dose calculation window | **Whole preg** | **T1** | **Whole preg** | **T1** |  | **Whole preg** | **T1** | **Whole preg** | **T1** |  |
| N | Number of births | **N = 381** | **N = 357** | **N = 484** | **N = 475** |  | **N = 430** | **N = 436** | **N = 435** | **N = 396** |  |
| Maternal age at delivery (years) | Mean [SD] | 30.9 (4.9) | 30.9 (4.9) | 31.5 (4.8) | 31.5 (4.7) | .099 | 31.1 (5.0) | 31.1 (4.9) | 31.4 (4.7) | 31.3 (4.7) | .404 |
|  | < 25 | 28 (7.3) | 26 (7.3) | 37 (7.6) | 34 (7.2) |  | 35 (8.1) | 33 (7.6) | 30 (6.9) | 27 (6.8) |  |
|  | 25-29 | 120 (31.5) | 114 (31.9) | 133 (27.5) | 132 (27.8) |  | 127 (29.5) | 132 (30.3) | 126 (29.0) | 114 (28.8) |  |
|  | 30-34 | 139 (36.5) | 131 (36.7) | 186 (38.4) | 184 (38.7) |  | 162 (37.7) | 163 (37.4) | 163 (37.5) | 152 (38.4) |  |
|  | ≥ 35 | 94 (24.7) | 86 (24.1) | 128 (26.4) | 125 (26.3) |  | 106 (24.7) | 108 (24.8) | 116 (26.7) | 103 (26.0) |  |
| Low-income status | CMU-C/C2S | 25 (6.6) | 25 (7.0) | 23 (4.8) | 20 (4.2) | .315 | 26 (6.0) | 26 (6.0) | 22 (5.1) | 19 (4.8) | .626 |
| Household social deprivation index (FDep) | Q1 (most favored) | 78 (20.5) | 77 (21.6) | 107 (22.1) | 103 (21.7) | .417 | 100 (23.3) | 105 (24.1) | 85 (19.5) | 75 (18.9) | .570 |
|  | Q2 | 88 (23.1) | 79 (22.1) | 93 (19.2) | 92 (19.4) |  | 87 (20.2) | 84 (19.3) | 94 (21.6) | 87 (22.0) |  |
|  | Q3 | 64 (16.8) | 58 (16.2) | 95 (19.6) | 94 (19.8) |  | 76 (17.7) | 77 (17.7) | 83 (19.1) | 75 (18.9) |  |
|  | Q4 | 81 (21.3) | 73 (20.4) | 89 (18.4) | 88 (18.5) |  | 77 (17.9) | 77 (17.7) | 93 (21.4) | 84 (21.2) |  |
|  | Q5 (least favored) | 64 (16.8) | 63 (17.6) | 87 (18.0) | 86 (18.1) |  | 80 (18.6) | 81 (18.6) | 71 (16.3) | 68 (17.2) |  |
|  | Unknown | 6 (1.6) | 7 (2.0) | 13 (2.7) | 12 (2.5) |  | 10 (2.3) | 12 (2.8) | 9 (2.1) | 7 (1.8) |  |
| Maternal estimated monthly income | Q1 income (0-€1825) | 41 (10.8) | 38 (10.6) | 47 (9.7) | 47 (9.9) | .584 | 40 (9.3) | 41 (9.4) | 48 (11.0) | 44 (11.1) | .934 |
|  | Q2 income (€1826-2286) | 41 (10.8) | 38 (10.6) | 64 (13.2) | 64 (13.5) |  | 55 (12.8) | 53 (12.2) | 50 (11.5) | 49 (12.4) |  |
|  | Q3 income (€2287-2745) | 46 (12.1) | 45 (12.6) | 49 (10.1) | 48 (10.1) |  | 45 (10.5) | 54 (12.4) | 50 (11.5) | 39 (9.8) |  |
|  | Q4 income (€2746-3504) | 59 (15.5) | 54 (15.1) | 63 (13.0) | 63 (13.3) |  | 61 (14.2) | 60 (13.8) | 61 (14.0) | 57 (14.4) |  |
|  | Q5 income (>€3504) | 69 (18.1) | 66 (18.5) | 85 (17.6) | 86 (18.1) |  | 79 (18.4) | 80 (18.3) | 75 (17.2) | 72 (18.2) |  |
|  | No income found | 125 (32.8) | 116 (32.5) | 176 (36.4) | 167 (35.2) |  | 150 (34.9) | 148 (33.9) | 151 (34.7) | 135 (34.1) |  |
| Maternal medical history | Hypertension | 12 (3.1) | 11 (3.1) | 13 (2.7) | 14 (2.9) | .842 | 13 (3.0) | 13 (3.0) | 12 (2.8) | 12 (3.0) | .977 |
|  | Diabetes | 1 (0.3) | 1 (0.3) | 3 (0.6) | 3 (0.6) | .635 | 2 (0.5) | 2 (0.5) | 2 (0.5) | 2 (0.5) | 1.000 |
|  | Psychiatric disorder | 6 (1.6) | 5 (1.4) | 8 (1.7) | 7 (1.5) | 1.000 | 5 (1.2) | 3 (0.7) | 9 (2.1) | 9 (2.3) | .420 |
|  | Psychotropic treatment | 63 (16.5) | 60 (16.8) | 80 (16.5) | 79 (16.6) | 1.000 | 68 (15.8) | 71 (16.3) | 75 (17.2) | 68 (17.2) | .636 |
|  | Multiple sclerosis | 5 (1.3) | 5 (1.4) | 0 (0.0) | 0 (0.0) | **.016** | 5 (1.2) | 5 (1.1) | 0 (0.0) | 0 (0.0) | **.030** |
|  | Narcolepsy | 81 (21.3) | 76 (21.3) | 167 (34.5) | 159 (33.5) | **<.001** | 116 (27.0) | 118 (27.1) | 132 (30.3) | 117 (29.5) | .308 |
|  | Sleep study | 108 (28.3) | 102 (28.6) | 95 (19.6) | 94 (19.8) | **.003** | 123 (28.6) | 123 (28.2) | 80 (18.4) | 73 (18.4) | **<.001** |
| Pregnancy-related characteristics | Gestational diabetes | 45 (11.8) | 43 (12.0) | 46 (9.5) | 44 (9.3) | .324 | 49 (11.4) | 51 (11.7) | 42 (9.7) | 36 (9.1) | .470 |
|  | Preeclampsia | 5 (1.3) | 4 (1.1) | 17 (3.5) | 16 (3.4) | **.050** | 11 (2.6) | 9 (2.1) | 11 (2.5) | 11 (2.8) | 1.000 |
|  | Folic acid suppl. | 177 (46.5) | 163 (45.7) | 207 (42.8) | 204 (42.9) | .310 | 222 (51.6) | 219 (50.2) | 162 (37.2) | 148 (37.4) | **<.001** |

Cohort baseline characteristics – dose-response analysis. DD daily dose, CD cumulated dose, CMU-C/C2S *Couverture maladie universelle complémentaire / Complémentaire santé solidaire*, FDep French social deprivation index, Q1-Q5 quintiles 1-5 of the distribution, LTD long-term disease, T1 first trimester of pregnancy, preg pregnancy

**Supplementary table D2**

|  | Prenatal exposure | Low DD modafinil  < 300 mg/day (T1) | High DD modafinil  ≥ 300 mg/day (T1) | p | Low CD modafinil  < 9.6 g (T1) | High CD modafinil  ≥ 9.6 g (T1) | P |
| --- | --- | --- | --- | --- | --- | --- | --- |
| N | Number of births | **N = 357** | **N = 457** |  | **N = 436** | **N = 396** |  |
| Issue | Live birth | 353 (98.9) | 467 (98.3) | .644 | 432 (99.1) | 388 (98.0) | .330 |
|  | Stillbirth | 2 (0.6) | 6 (1.3) |  | 3 (0.7) | 5 (1.3) |  |
|  | Medical termination | 2 (0.6) | 2 (0.4) |  | 1 (0.2) | 3 (0.8) |  |
| MCA | MCA | 18 (5.0) | 17 (3.6) | .386 | 20 (4.6) | 15 (3.8) | .856 |

Birth outcomes – dose-response analysis, based on first-trimester exposure. DD daily dose, CD cumulated dose, MCA major congenital anomaly, T1 first trimester of pregnancy

**Supplementary table D3**

|  | Prenatal exposure | Low DD modafinil  < 300 mg/day  (whole pregnancy) | High DD modafinil  ≥ 300 mg/day  (whole pregnancy) | p | Low CD modafinil  < 10 g  (whole pregnancy) | High CD modafinil  ≥ 10 g  (whole pregnancy) | p |
| --- | --- | --- | --- | --- | --- | --- | --- |
| N | Number of live births | **N = 377** | **N = 476** |  | **N = 426** | **N = 427** |  |
| Delivery | Cesarean section | 89 (23.6) | 98 (20.6) | .329 | 96 (22.5) | 91 (21.3) | .727 |
|  | Gestational age, Mean [SD] | 39.2 [1.7] | 39.2 [1.7] | .966 | 39.2 [1.8] | 39.2 [1.7] | .825 |
|  | Prematurity (< 37 WG) | 19 (5) | 21 (4.4) | .789 | 18 (4.2) | 22 (5.2) | .632 |
|  | Preterm (32-36 WG) | 17 (4.5) | 19 (4) |  | 15 (3.5) | 21 (4.9) |  |
|  | Very preterm (28-31 WG) | 1 (0.3) | 1 (0.2) |  | 2 (0.5) | 0 (0) |  |
|  | Extremely preterm (< 28 WG) | 1 (0.3) | 1 (0.2) |  | 1 (0.2) | 1 (0.2) |  |
| Infant characteristics | Sex M | 214 (56.8) | 244 (51.3) | .126 | 230 (54) | 228 (53.4) | .916 |
|  | Birth weight (g), Mean [SD] | 3,308 [493] | 3,305 [488] | .924 | 3,305 [491] | 3,308 [489] | .942 |
|  | SGA | 31 (8.2) | 44 (9.2) | .688 | 36 (8.5) | 39 (9.1) | .817 |
| ND [IRR/1000PY] | Any ND | 13 [3.6] | 15 [3.5] | .943 | 16 [4] | 12 [3.1] | .495 |
|  | Intellectual disability | 1 [0.3] | 2 [0.5] | .671 | 3 [0.7] | 0 [0] | .095 |
|  | Disorders of psych. development | 10 [2.8] | 13 [3] | .822 | 13 [3.2] | 10 [2.6] | .576 |
|  | Behavioral and emot. disorders | 6 [1.6] | 6 [1.4] | .752 | 7 [1.7] | 5 [1.3] | .600 |
| Specialized consultations [IRR/1000PY] | Any specialized consultation | 141 [48.3] | 152 [41.9] | .260 | 153 [46.5] | 140 [43] | .493 |
|  | Speech therapy | 120 [39.2] | 140 [37.7] | .847 | 133 [38.8] | 127 [38] | .840 |
|  | Psychiatrist | 44 [12.9] | 27 [6.3] | **.003** | 44 [11.4] | 27 [7.1] | .054 |
|  | Psychoeducational center | 1 [0.3] | 2 [0.5] | .684 | 2 [0.5] | 1 [0.3] | .580 |

Obstetric, fetal and pediatric outcomes, restricted to live births – dose-response analysis, based on exposure at any point during pregnancy. DD daily dose, CD cumulated dose, WG weeks of gestation, g grams, SD standard deviation, SGA small for gestational age, ND neurodevelopmental disorder, IRR/1000PY incidence rate ratio per 1000 persons-years

# [Appendix](https://www.ncbi.nlm.nih.gov/pmc/articles/PMC10781191/) E: First sensitivity analysis (at least two dispensations during the first trimester of pregnancy)

**Supplementary table E1**

|  | Prenatal exposure | Modafinil  (≥ 2 dispensations at T1) | Methylphenidate  (≥ 2 dispensations at T1) | Unexposed | PS-matched unexposed  (pairs with ≥ 2 disp. at T1) | p_1_ | p_2_ |
| --- | --- | --- | --- | --- | --- | --- | --- |
| N | Number of births | **N = 405** | **N = 380** | **N = 10,953,951** | **N = 405** | **.030** | .849 |
| Maternal age at delivery (years) | Mean [SD] | 31.0 (4.8) | 31.9 (5.8) | 1,640,149 (15.0) | 31.1 (4.8) |  |  |
|  | < 25 | 30 (7.4) | 43 (11.3) | 3,348,073 (30.6) | 30 (7.4) |  |  |
|  | 25-29 | 125 (30.9) | 71 (18.7) | 3,613,924 (33.0) | 120 (29.6) |  |  |
|  | 30-34 | 149 (36.8) | 130 (34.2) | 2,351,805 (21.5) | 160 (39.5) |  |  |
|  | ≥ 35 | 101 (24.9) | 136 (35.8) | 1,787,542 (16.3) | 95 (23.5) |  |  |
| Low-income status | CMU-C/C2S beneficiaries | 19 (4.7) | 61 (16.1) | 2,089,610 (19.1) | 26 (6.4) | **<.001** | 0.357 |
| Household social deprivation index (FDep) | Q1 (most favored) | 86 (21.2) | 100 (26.3) | 2,084,855 (19.0) | 79 (19.5) | .108 | .866 |
|  | Q2 | 86 (21.2) | 88 (23.2) | 2,094,955 (19.1) | 79 (19.5) |  |  |
|  | Q3 | 71 (17.5) | 76 (20.0) | 2,088,057 (19.1) | 83 (20.5) |  |  |
|  | Q4 | 85 (21.0) | 66 (17.4) | 2,094,421 (19.1) | 81 (20.0) |  |  |
|  | Q5 (least favored) | 68 (16.8) | 46 (12.1) | 502,053 (4.6) | 74 (18.3) |  |  |
|  | Unknown | 9 (2.2) | 4 (1.1) | 1,227,199 (11.2) | 9 (2.2) |  |  |
| Maternal estimated monthly income | Q1 income (0-€1825) | 45 (11.1) | 29 (7.6) | 1,227,172 (11.2) | 43 (10.6) | **.019** | .933 |
|  | Q2 income (€1826-2286) | 52 (12.8) | 40 (10.5) | 1,227,182 (11.2) | 45 (11.1) |  |  |
|  | Q3 income (€2287-2745) | 46 (11.4) | 52 (13.7) | 1,227,159 (11.2) | 54 (13.3) |  |  |
|  | Q4 income (€2746-3504) | 58 (14.3) | 50 (13.2) | 1,227,116 (11.2) | 58 (14.3) |  |  |
|  | Q5 income (>€3504) | 72 (17.8) | 47 (12.4) | 4,818,123 (44.0) | 69 (17.0) |  |  |
|  | No income found | 132 (32.6) | 162 (42.6) | 121,032 (1.1) | 136 (33.6) |  |  |
| Maternal medical history | Hypertension | 15 (3.7) | 13 (3.4) | 69,332 (0.6) | 14 (3.5) | .983 | 1.000 |
|  | Diabetes | 2 (0.5) | 5 (1.3) | 29,706 (0.3) | 5 (1.2) | .273 | .451 |
|  | Psychiatric disorder | 5 (1.2) | 24 (6.3) | 396,016 (3.6) | 5 (1.2) | **<.001** | 1.000 |
|  | Psychotropic treatment | 72 (17.8) | 151 (39.7) | 9,115 (0.1) | 74 (18.3) | **<.001** | .927 |
|  | Multiple sclerosis | 1 (0.2) | 0 (0.0) | 1,298 (<0.1) | 1 (0.2) | 1.000 | 1.000 |
|  | Narcolepsy | 115 (28.4) | 40 (10.5) | 21,911 (0.2) | 115 (28.4) | **<.001** | 1.000 |
|  | Sleep study | 90 (22.2) | 30 (7.9) | 1,344,494 (12.3) | 94 (23.2) | **<.001** | .801 |
| Pregnancy-related characteristics | Gestational diabetes | 42 (10.4) | 54 (14.2) | 208,639 (1.9) | 71 (17.5) | .125 | **.005** |
|  | Preeclampsia | 12 (3.0) | 12 (3.2) | 4,137,928 (37.8) | 13 (3.2) | 1.000 | 1.000 |
|  | Folic acid supplementation | 153 (37.8) | 173 (45.5) | 3,348,073 (30.6) | 186 (45.9) | **.033** | **.023** |

Cohort baseline characteristics – sensitivity analysis. PS propensity score, SD standard deviation, CMU-C/C2S *Couverture maladie universelle complémentaire / Complémentaire santé solidaire*, FDep French social deprivation index, Q1-Q5 quintiles 1-5 of the distribution, LTD long-term disease, T1 first trimester of pregnancy

p_1_: Modafinil *vs*. Methylphenidate, p_2_: Modafinil *vs*. PS-matched unexposed

**Supplementary table E2**

|  | Prenatal exposure | Modafinil  (≥ 2 dispensations at T1) | Methylphenidate  (≥ 2 dispensations at T1) | Unexposed | PS-matched unexposed  (pairs with ≥ 2 disp. at T1) | p_1_ | p_2_ |
| --- | --- | --- | --- | --- | --- | --- | --- |
| N | Number of births | **N = 405** | **N = 380** | **N = 10,953,951** | **N = 405** |  |  |
| Issue | Live birth | 399 (98.5) | 375 (98.7) | 10,871,565 (99.2) | 401 (99.0) | 1.000 | .706 |
|  | Stillbirth | 3 (0.7) | 2 (0.5) | 49,832 (0.5) | 3 (0.7) |  |  |
|  | Medical termination | 3 (0.7) | 3 (0.8) | 32,554 (0.3) | 1 (0.2) |  |  |
| MCA | **MCA** | 14 (3.5) | 10 (2.6) | 328,784 (3.0) | 10 (2.5) | .643 | .534 |

Birth outcomes – sensitivity analysis. PS propensity score, MCA major congenital anomaly, T1 first trimester of pregnancy

p_1_: Modafinil *vs*. Methylphenidate, p_2_: Modafinil *vs*. PS-matched unexposed

# [Appendix](https://www.ncbi.nlm.nih.gov/pmc/articles/PMC10781191/) F: Second sensitivity analysis (restricted to term births)

**Supplementary table F1**

|  | Prenatal exposure | Modafinil  (whole pregnancy) | Methylphenidate  (whole pregnancy) | Unexposed | PS-matched unexposed | p_1_ | p_2_ |
| --- | --- | --- | --- | --- | --- | --- | --- |
| N | Number of births | **N = 813** | **N = 870** | **N = 10,283,788** | **N = 806** |  |  |
| Maternal age at delivery (years) | Mean [SD] | 31.2 (4.8) | 32.6 (5.4) | 30.2 (5.3) | 31.3 (4.9) | **<.001** | .756 |
|  | < 25 | 62 (7.6) | 72 (8.3) | 1,522,979 (14.8) | 60 (7.4) |  |  |
|  | 25-29 | 238 (29.3) | 149 (17.1) | 3,153,965 (30.7) | 228 (28.3) |  |  |
|  | 30-34 | 309 (38.0) | 299 (34.4) | 3,414,423 (33.2) | 317 (39.3) |  |  |
|  | ≥ 35 | 204 (25.1) | 350 (40.2) | 2,192,421 (21.3) | 201 (24.9) |  |  |
| Low-income status | CMU-C/C2S beneficiaries | 44 (5.4) | 176 (20.2) | 1,644,654 (16.0) | 51 (6.3) | **<.001** | .498 |
| Household social deprivation index (FDep) | Q1 (most favored) | 174 (21.4) | 209 (24.0) | 1,974,459 (19.2) | 172 (21.3) | .230 | .998 |
|  | Q2 | 174 (21.4) | 186 (21.4) | 1,964,899 (19.1) | 167 (20.7) |  |  |
|  | Q3 | 151 (18.6) | 178 (20.5) | 1,971,741 (19.2) | 154 (19.1) |  |  |
|  | Q4 | 156 (19.2) | 166 (19.1) | 1,960,408 (19.1) | 156 (19.4) |  |  |
|  | Q5 (least favored) | 141 (17.3) | 115 (13.2) | 1,956,791 (19.0) | 138 (17.1) |  |  |
|  | Unknown | 17 (2.1) | 16 (1.8) | 455,490 (4.4) | 19 (2.4) |  |  |
| Maternal estimated monthly income | Q1 income (0 - €1825) | 77 (9.5) | 89 (10.2) | 1,143,425 (11.1) | 78 (9.7) | **.002** | .994 |
|  | Q2 income (€1826 - €2286) | 104 (12.8) | 91 (10.5) | 1,155,321 (11.2) | 96 (11.9) |  |  |
|  | Q3 income (€2287 - €2745) | 89 (10.9) | 96 (11.0) | 1,162,257 (11.3) | 92 (11.4) |  |  |
|  | Q4 income (€2746 - €3504) | 115 (14.1) | 98 (11.3) | 1,164,770 (11.3) | 111 (13.8) |  |  |
|  | Q5 income (> €3504) | 146 (18.0) | 117 (13.4) | 1,167,015 (11.3) | 144 (17.9) |  |  |
|  | No income found | 282 (34.7) | 379 (43.6) | 4,491,000 (43.7) | 285 (35.4) |  |  |
| Maternal medical history | Hypertension | 22 (2.7) | 23 (2.6) | 104,181 (1.0) | 24 (3.0) | 1.000 | .858 |
|  | Diabetes | 3 (0.4) | 8 (0.9) | 56,114 (0.5) | 11 (1.4) | .228 | **.034** |
|  | Psychiatric disorder | 13 (1.6) | 32 (3.7) | 27,001 (0.3) | 9 (1.1) | **.013** | .533 |
|  | Psychotropic treatment | 128 (15.7) | 245 (28.2) | 363,778 (3.5) | 132 (16.4) | **<.001** | .780 |
|  | Multiple sclerosis | 5 (0.6) | 4 (0.5) | 8,492 (0.1) | 2 (0.2) | .746 | .452 |
|  | Narcolepsy | 232 (28.5) | 63 (7.2) | 1,178 (<0.1) | 226 (28.0) | **<.001** | .868 |
|  | Sleep study | 198 (24.4) | 59 (6.8) | 20,124 (0.2) | 195 (24.2) | **<.001** | .986 |
| Pregnancy-related characteristics | Gestational diabetes | 87 (10.7) | 135 (15.5) | 1,250,390 (12.2) | 122 (15.1) | **.004** | **.010** |
|  | Preeclampsia | 19 (2.3) | 18 (2.1) | 126,314 (1.2) | 14 (1.7) | .835 | .497 |
|  | Folic acid supplementation | 363 (44.6) | 399 (45.9) | 3,882,711 (37.8) | 359 (44.5) | .652 | 1.000 |

Cohort baseline characteristics, restricted to live term births, based on exposure at any point during pregnancy. PS propensity score, SD standard deviation, CMU-C/C2S *Couverture maladie universelle complémentaire / Complémentaire santé solidaire*, FDep French social deprivation index, Q1-Q5 quintiles 1-5 of the distribution, LTD long-term disease, T1 first trimester of pregnancy

p_1_: Modafinil *vs*. Methylphenidate, p_2_: Modafinil *vs*. PS-matched unexposed

**Supplementary table F2**

|  | Prenatal exposure | Modafinil  (whole pregnancy) | Methylphenidate (whole pregnancy) | Unexposed | PS-matched unexposed (all pairs) | p_1_ | p_2_ |
| --- | --- | --- | --- | --- | --- | --- | --- |
| N | Number of live births | **N = 813** | **N = 867** | **N = 10,270,386** | **N = 802** |  |  |
| Delivery | Cesarean section | 172 (21.2) | 212 (24.5) | 1,927,142 (18.8) | 166 (20.7) | .121 | .869 |
|  | Gestational age, Mean [SD] | 39.5 (1.2) | 39.2 (1.1) | 39.4 (1.2) | 39.5 (1.2) | **<.001** | .976 |
| Infant characteristics | Sex M | 436 (53.6) | 448 (51.7) | 5,228,094 (50.9) | 412 (51.4) | .451 | .391 |
|  | Birth weight (g), Mean [SD] | 3,350 (440) | 3,341 (455) | 3,341 (448) | 3,344 (441) | .680 | .766 |
|  | SGA | 74 (9.1) | 75 (8.7) | 1,008,208 (9.8) | 75 (9.4) | .811 | .930 |
| ND [IRR/1000PY] | Any ND | 25 [3.3] | 50 [9.4] | 247,339 [2.7] | 23 [3.3] | **<.001** | .993 |
|  | Intellectual disability | 3 [0.4] | 6 [1.1] | 33,147 [0.4] | 3 [0.4] | .125 | .912 |
|  | Disorders of psych. development | 20 [2.6] | 33 [6.1] | 163,586 [1.8] | 13 [1.8] | **.001** | .319 |
|  | Behavioral and emot. disorders | 11 [1.4] | 22 [4.0] | 104,446 [1.1] | 13 [1.8] | **.001** | .521 |
| Specialized consultations  [IRR/1000PY] | Any specialized consultation | 273 [43.5] | 246 [54.8] | 2,585,654 [32.6] | 232 [39.4] | **<.001** | .310 |
|  | Speech therapy | 240 [36.9] | 206 [44.1] | 2,352,125 [29.1] | 212 [35.1] | **.003** | .638 |
|  | Psychiatrist | 68 [9.3] | 77 [14.9] | 452,586 [5.0] | 45 [6.5] | **<.001** | .073 |
|  | Psychoeducational center | 3 [0.4] | 3 [0.5] | 42,017 [0.5] | 4 [0.6] | .594 | .622 |

Obstetric, fetal and pediatric outcomes based on exposure at any point during pregnancy, restricted to live term births, based on exposure at any point during pregnancy. PS propensity score, WG weeks of gestation, g grams, SD standard deviation, SGA small for gestational age, ND neurodevelopmental disorder, IRR/1000PY incidence rate ratio per 1000 persons

p_1_: Modafinil *vs*. Methylphenidate, p_2_: Modafinil *vs*. PS-matched unexposed

**Supplementary figure F1**

**
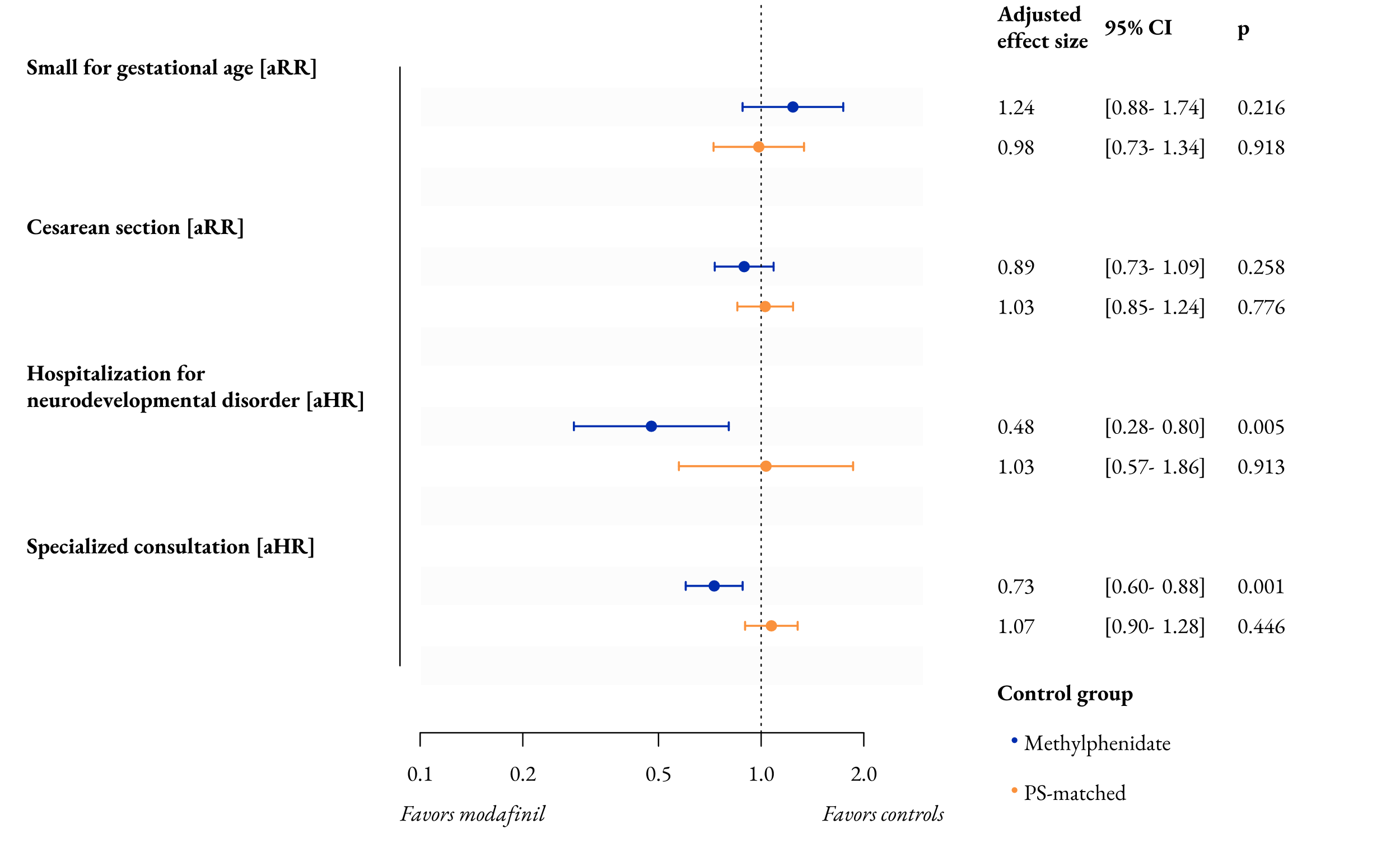
**

Adjusted risk ratios and hazard ratios of outcomes based on exposure at any point during pregnancy, restricted to live term births. CI confidence interval, RR risk ratio, HR hazard ratio, PS propensity score
